# m^6^A‐Dependent ITIH1 Regulated by TGF‐β Acts as a Target for Hepatocellular Carcinoma Progression

**DOI:** 10.1002/advs.202401013

**Published:** 2024-09-05

**Authors:** Zhibin Liao, Hongwei Zhang, Furong Liu, Weijian Wang, Yachong Liu, Chen Su, He Zhu, Xiaoping Chen, Bixiang Zhang, Zhanguo Zhang

**Affiliations:** ^1^ Hepatic Surgery Center Tongji Hospital Tongji Medical College Huazhong University of Science and Technology Wuhan Hubei 430030 China; ^2^ Hubei Key Laboratory of Hepato‐Pancreato‐Biliary Diseases Wuhan Hubei 430030 China; ^3^ Key Laboratory of Organ Transplantation Ministry of Education and Ministry of Health Wuhan Hubei 430030 China

**Keywords:** hepatocellular carcinoma, integrin/FAK signaling, ITIH1, m^6^A, TGF‐β

## Abstract

Both the transforming growth factor beta (TGF‐β) signaling pathway and N6‐methyladenosine (m^6^A) modification for mRNA play an important role in hepatocellular carcinoma (HCC) progression. However, the relationship between TGF‐β and m^6^A in hepatocellular carcinoma (HCC) remains unclear. Here, it is found that TGF‐β can promote the liquid phase separation of METTL3, which further leads to the reduction of mRNA stability of ITIH1. As a secreted protein, ITIH1 can act as a ligand of integrin α5β1 to antagonize fibronectin, induce the inhibition of focal adhesion kinase signaling pathway, and inhibit the progression of HCC. In the preclinical model (mouse model, patient‐derived organoid, patient‐derived xenografts), purified recombinant ITIH1 (r‐ITIH1) protein can be targeted for HCC. More importantly, r‐ITIH1 can play a synergistic role in targeting HCC with TGF‐β inhibitor. The downstream ITIH1 regulatory mechanism of TGF‐β and m^6^A modification is revealed, and ITIH1 can be translational as a potential target for HCC.

## Graphical abstract

This study uncovers that ITIH1, as a secreted protein in HCC, acts as a ligand to antagonize fibronectin, leading to the inhibition of the FAK signaling pathway to attenuate the progression of HCC. Integrating the preclinical models, this work paves the way for the purified recombinant ITIH1 as a potential therapeutic target for HCC.

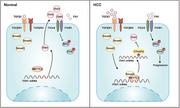

## 1 Introduction

Hepatocellular carcinoma (HCC) is a kind of the most familiar malignancies worldwide. It is the sixth most frequently diagnosed malignancy and the fourth leading cause of cancer‐related death.^[^ ^1^ ^]^ Although great efforts have been made to develop therapeutic strategies, including surgical resection, liver transplantation, new therapeutic drugs, and comprehensive therapy, over the past decades, the Five year survival rate remains unsatisfactory.^[^ ^2^, ^3^, ^4^ ^]^ Hence, elucidating the molecular mechanisms underlying HCC and identifying novel therapeutic options are essential for HCC treatment.

Transforming growth factor beta (TGF‐β) is a multifunctional cytokine that is involved in the pathogenesis of chronic liver diseases and cancers.^[^ ^5^, ^6^, ^7^ ^]^ Activation of the canonical TGF‐β signaling begins with the formation of a heterotetrameric complex of type I and type II serine/threonine kinase receptors (TGFBR1/2) after TGF‐β binding. The subsequent phosphorylation of the c‐termini of SMAD2 and SMAD3 (R‐SMADs) leads to the formation of a complex with SMAD4 (co‐SMAD). These SMAD complexes then translocate into the nucleus to control the transcription of many downstream target genes.^[^ ^8^, ^9^, ^10^ ^]^ Accordingly, targeted compounds that inhibit TGFBR1 function have been developed to suppress HCC progression. Galunisertib, a TGFBR1 inhibitor, has been used clinically to improve the overall survival (OS) of HCC patients.^[^ ^11^, ^12^, ^13^, ^14^ ^]^ Additionally, SMAD2/3 has been reported to promote the recruitment of the m^6^A methyltransferase complex to a subset of transcripts which were involved in early cell fate decisions, highlighting a function beyond their role in transcription.^[^ ^15^ ^]^

Reversible RNA modifications introduce a new level of posttranscriptional regulation of gene expression, engaging in many physiological and pathological processes.^[^ ^16^, ^17^, ^18^ ^]^ N6‐methyladenosine (m^6^A) is a dynamic and reversible chemical modification that is the most prevalent in mRNA.^[^ ^19^ ^]^ It broadly influences mRNA metabolism, impacting mRNA degradation, stability, and translation.^[^ ^20^, ^21^ ^]^ Methyltransferase‐like 3 (METTL3), a key component of the “writer” complex (comprising METTL3, METTL14, and WTAP), plays a vital role in gene expression regulation. It has been shown that METTL3 could interact with itself, providing multivalency to form condensation that regulates the activity of the m^6^A methyltransferase complex. Furthermore, numerous studies have revealed that METTL3 dysfunction accelerates tumor growth, metastasis, and drug resistance in various human cancers.^[^ ^22^, ^23^, ^24^ ^]^ m^6^A mRNA modification is started by “writers” and then recognized by “readers,” which preferentially bind to an RR (m^6^A) CU (R = G or A) consensus motif. “Readers” contain YTH domains that specifically bind to m6A‐modified RNA and regulate mRNA splicing, degradation, stability, and translation.^[^ ^25^, ^26^ ^]^ The mechanisms through which m^6^A regulates gene expression need further research in HCC.

Integrin signaling is involved in many cellular functions, such as adhesion, migration, and oncogenic transformation.^[^ ^27^, ^28^ ^]^ Aberrant activation of integrin signaling has been linked with the malignant features of HCC.^[^ ^29^ ^]^ One of the most important downstream effectors is focal adhesion kinase (FAK), which has been shown to drive HCC progression and correlate with a poor clinical outcome. Activated FAK kinase can further phosphorylate and activate downstream signaling molecules, including Src and AKT.^[^ ^30^ ^]^

Extracellular matrix (ECM) proteins can function as ligands and regulators of the integrin/FAK signaling pathway and are thus involved in cancer progression.^[^ ^31^ ^]^ Inter‐alpha (globulin) inhibitor H1 (ITIH1) is a member of a heavy chain family, whose currently known function of the heavy chains is to provide a covalent linkage to hyaluronic acid, which is a major component of the ECM.^[^ ^32^ ^]^ In this study, we explored the mechanisms by which ITIH1 regulates HCC development and tested its therapeutic potential in HCC in mice.

## 2 Results

### 2.1 ITIH1 is Downregulated by TGF‐β Stimulation

**Figure 1:**
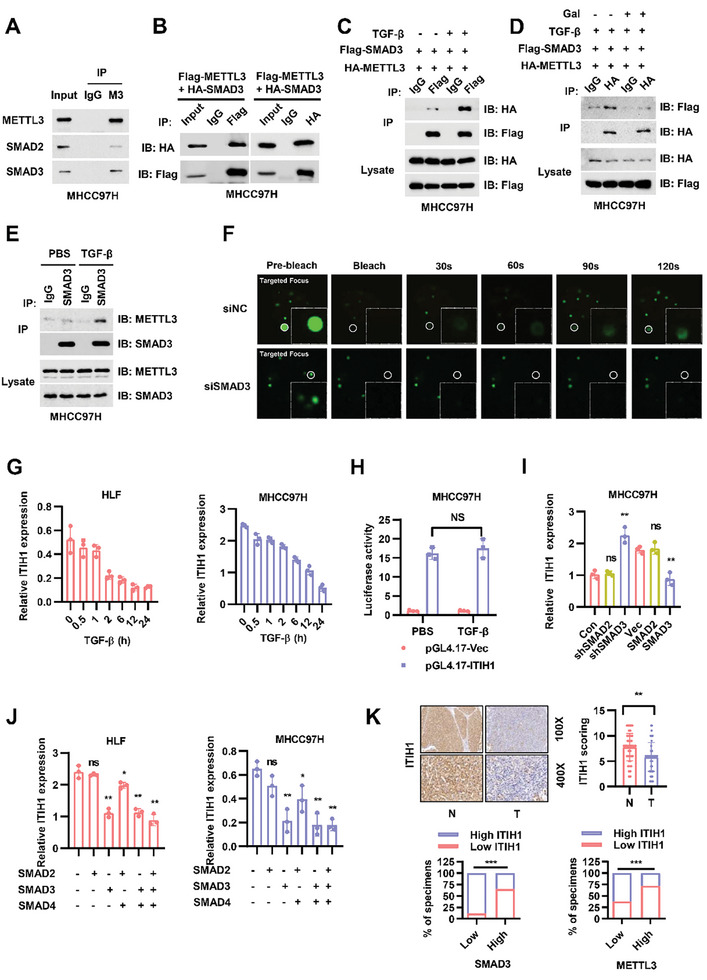
ITIH1 is downregulated by TGF‐β stimulation. A) The results of METTL3 and SMAD2/3 co‐IP in MHCC97H cells. B) The results of Flag‐SMAD3 and HA‐METTL3 co‐IP in MHCC97H cells. C) The results of Flag‐SMAD3 and HA‐METTL3 co‐IP in MHCC97H cells with or without TGF‐β stimulation for 12 h. D) The results of Flag‐SMAD3 and HA‐METTL3 co‐IP with stimulation for 12 h and with galunisertib for 12 h in MHCC97H cells. E) The results of SMAD3 and METTL3 endogenous co‐IP with or without TGF‐β stimulation for 12 h in MHCC97H cells. F) MHCC97H cells with exogenous expression of GFP‐METTL3 undergo liquid‐like behavior; fluorescence recovery after photobleaching (FRAP) images are shown before and at indicated time points after bleaching. Quantification of fluorescence intensity recovery of GFP‐METTL3 in the bleached droplet. G) The relative time‐dependent ITIH1 mRNA expression in HLF and MHCC97H cells with TGF‐β stimulation. H) The relative luciferase activity in MHCC97H cells transfected with pGL4.17 plasmids containing the ITIH1 promoter region with TGF‐β stimulation for 12 h. I) The relative ITIH1 mRNA expression when SMAD2 or SMAD3 was knocked down or overexpressed in MHCC97H cells. J) The relative ITIH1 mRNA expression when HLF and MHCC97H cells were transfected with the indicated plasmids. K) Representative results of IHC staining for ITIH1 in paraffin‐embedded HCC samples from Tongji Hospital. The statistical results of their expression in nontumor tissues (N) and tumor tissues (T) are presented in the right panel. The lower graphs show the correlations between ITIH1 and METTL3/SMAD3 expression as determined from the IHC results. Data are represented as means ± SD in the bar graphs. ns: not significant, ^*^: *p* < 0.05, ^**^: *p* < 0.01, ^***^: *p* < 0.001. M3, METTL3; Gal, galunisertib.

It has been reported that the interaction of SMAD2/3 with m^6^A writer complexes promotes m^6^A deposition on a subset of transcripts.^[^ ^15^ ^]^ We then confirmed the binding of SMAD3 and METTL3, one of the important components of m^6^A writer complexes, in HCC progression (**Figure** **1**A,B; Figure S1A, Supporting Information left panel). TGF‐β induced activation of SMAD complexes then further enhanced the association between SMAD3 and METTL3 (Figure 1C); on the contrary, galunisertib (LY2157299, a selective inhibitor of TGFβR1) treatment led to decreased binding capacity (Figure 1D). This result was verified by an endogenous IP assay (Figure 1E; Figure S1A, Supporting Information right panel). METTL3, as well as other members of m^6^A methyltransferase complexes, undergoes liquid–liquid phase separation, and the METTL3 condensate is closely related to the functions of METTL3.^[^ ^33^ ^]^ FRAP and fusion/fission assays revealed that METTL3 puncta underwent phase separation and the knockdown of SMAD3 attenuated the formation of METTL3 condensates (Figure 1F; Figure S1F,G, Supporting Information), suggesting that SMAD3 might promote the formation of METTL3 condensates, which results in stronger functional regulation of writer complexes. In turn, the crucial proteins mediating m^6^A modification (METTL3 and ALKBH5) were not able to alter the activation of the TGF‐β signaling pathway (Figure S1B, Supporting Information). The results revealed that TGF‐β stimulation promoted the combination between SMAD3 and METTL3, and SMAD3 was indispensable for the formation of METTL3 condensates.

To further explore how SMAD3 and METTL3 regulate HCC progression, we used data from RNA sequencing (RNA‐seq) in MHCC97H cells with SMAD3 or METTL3 knockdown (Figure S1C, Supporting Information). These twelve genes regulated by both SMAD3 and METTL3 were then verified through qRT–PCR in MHCC97H cells (Figure S1D,E, Supporting Information). ITIH1 appeared to be the top hit due to its simultaneous regulation by SMAD3 and METLL3. In HLF and MHCC97H cells, we found that the ITIH1 mRNA level was decreased upon TGF‐β stimulation in a time‐dependent manner (Figure 1G). As SMAD proteins act as transcriptional complexes to regulate the expression of downstream targets, we also used reporter assay to examine whether ITIH1 is transcriptionally regulated by SMAD3. The results showed that TGF‐β stimulation did not affect the transcription of ITIH1, indicating that decreased expression of ITIH1 upon SMAD3 knockdown was independent of its transcriptional activity (Figure 1H). It is therefore possible that TGF‐β regulated ITIH1 at the posttranscriptional level. To explore the roles of SMAD proteins, we tested the effects of ectopic expression of SMAD2/3/4, and the results showed that SMAD3 was indispensable for the downregulation of ITIH1 caused by TGF‐β (Figure 1I,J). According to the GEPIA (http://gepia.cancer‐pku.cn/) prediction results, ITIH1 expression was negatively correlated with the expression of SMAD3 and METTL3 in HCC samples (Figure S2A,B, Supporting Information). The IHC staining of the three proteins on paraffin‐embedded HCC tissues acquired from Tongji Hospital further confirmed a negative correlation between ITIH1 and SMAD3/METTL3 (Figure 1K; Figure S2C,D, Supporting Information).

The above results indicated that SMAD3 might promote the formation of METTL3 condensates and regulate the *ITIH1* mRNA level.

### 2.2 METTL3 Regulates ITIH1 Expression in an m^6^A ‐Dependent Manner

**Figure 2:**
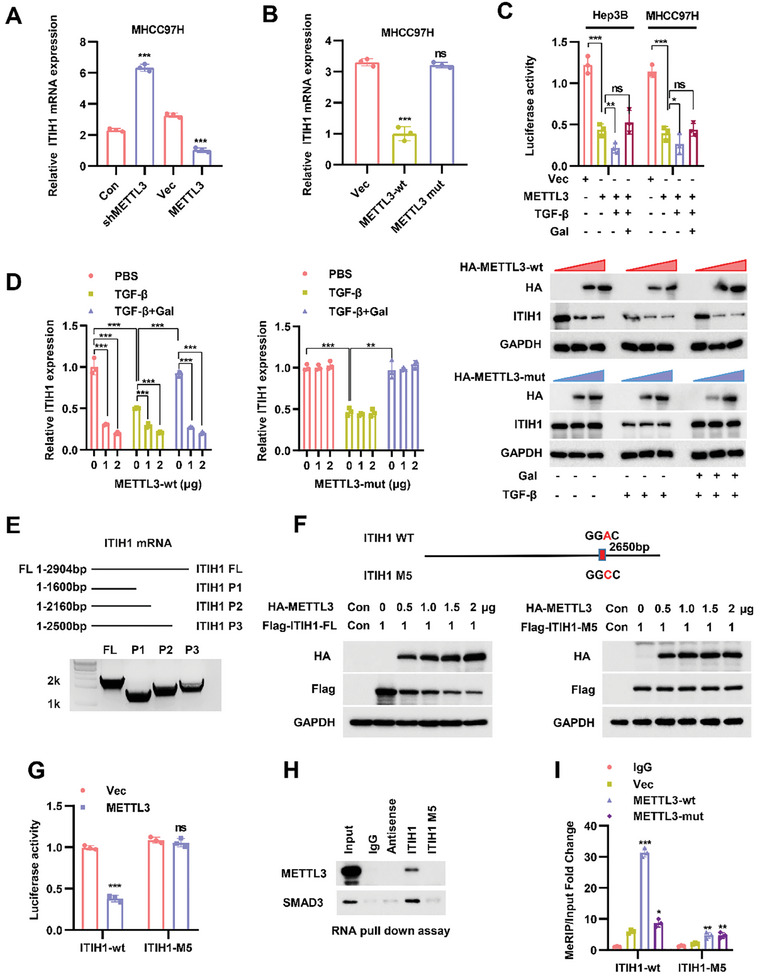
METTL3 regulates the expression of ITIH1 in an m^6^A‐dependent manner. A) The relative ITIH1 mRNA expression when METTL3 was knocked down or overexpressed in MHCC97H cells. B) The relative ITIH1 expression in MHCC97H cells transfected separately with the METTL3‐wt and METTL3‐mut (catalytic point mutation) plasmids. C) The relative luciferase activity in Hep3B and MHCC97H cells cotransfected with psiCHECK2 containing the ITIH1 mRNA sequence and a METTL3 plasmid and then stimulated with TGF‐β or galunisertib. D) The relative ITIH1 mRNA expression (left two panels) and ITIH1 protein levels (rightmost panel) when 293T cells were transfected with METTL3‐wt or METTL3‐mut in a concentration‐dependent manner and then stimulated with TGF‐β or galunisertib. E) Schematic diagram of the ITIH1 truncation mutants. F) The protein levels in 293T cells cotransfected with HA‐METTL3 and Flag‐ITIH1‐FL (full length)/M5 in a concentration‐dependent manner. G) The relative luciferase activity in cells transfected with psiCHECK2 containing ITIH1‐wt or ITIH1‐M5 and overexpressing METTL3‐WT in 293T cells. H) Results of RNA pulldown with ITIH1‐wt or ITIH1‐M5 in 293T cells. I) MeRIP results in 293T cells cotransfected with ITIH1‐M5 and METTL3‐Mut. Data are represented as means ± SD in the bar graphs. ns: not significant, ^*^: *p* < 0.05, ^**^: *p* < 0.01, ^***^: *p* < 0.001. Gal, galunisertib.

To figure out how METTL3 regulates ITIH1 expression, we tested the relative mRNA level of ITIH1 when METTL3 was knocked down or overexpressed in MHCC97H cells. The results reflected that ITIH1 expression was negatively controlled by METTL3 (**Figure** **2**A). Since METTL3 is an important “writer” of the m^6^A modification process, inactive METTL3‐mut was taken advantage of to test whether METTL3‐mediated ITIH1 regulation is m^6^A dependent. When METTL3‐mut was expressed, the *ITIH1* mRNA level was not affected (Figure 2B). Then, we constructed a psiCHECK2 plasmid containing the *ITIH1* sequence. The dual luciferase reporter assay suggested that METTL3 might bind to *ITIH1* mRNA. This process was enhanced by TGF‐β and inhibited by galunisertib (Figure 2C). These data indicated that METTL3 might regulate ITIH1 in an m^6^A ‐dependent manner. Moreover, METTL3 downregulated ITIH1 expression in a dose‐dependent manner with TGF‐β enhancing this process. Nevertheless, TGF‐β stimulation can no longer affect ITIH1 expression when inactivated METTL3‐mut is expressed (Figure 2D). According to the SRAMP (http://www.cuilab.cn/sramp) analysis, four predicted m^6^A sites (very high confidence) were identified in *ITIH1* mRNA (Figure S3A, Supporting Information). To confirm whether the four sites are responsible for METTL3 regulation, point mutations were introduced into the putative m^6^A site: *ITIH1*‐M1, *ITIH1*‐M2, *ITIH1*‐M3, and *ITIH1*‐M4. When cotransfected with an increasing dose of METTL3, no change was found for ITIH1 mutant levels (Figure S4A, Supporting Information). Next, a truncation mutant of *ITIH1* was applied to confirm that the m^6^A modification site is located between 2500 and 2904 bp (Figure 2E; Figure S4B, Supporting Information). However, another m^6^A site (high confidence) at 2650 bp was also found confirmed by using the same strategy (*ITIH1*‐M5) (Figure 2F,G; Figure S3B, Supporting Information). RNA pulldown assay further supported that METTL3 and SMAD3 are no longer bound to *ITIH1*‐M5 mRNA (Figure 2H). The Me‐RIP assay showed that the METTL3‐mediated m^6^A modification in *ITIH1*‐M5 was much lower than that in *ITIH1*‐wt (Figure 2I).

These data together revealed that METTL3 catalyzes m^6^A modification on *ITIH1* mRNA which might related to the regulation of its expression.

### 2.3 YTHDF2 Facilitates the Decay of *ITIH1* mRNA in an m^6^A ‐Dependent Manner

**Figure 3:**
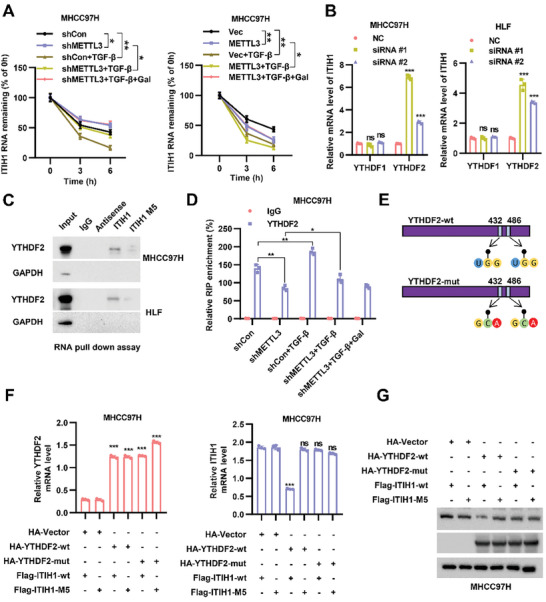
YTHDF2 facilitates the decay of ITIH1 mRNA in an m^6^A‐dependent manner. A) Levels of remaining ITIH1 mRNA in MHCC97H‐shMETTL3 cells (left) and MHCC97H‐oeMETTL3 cells (right) treated with TGF‐β, galunisertib, and actinomycin D for the indicated times. B) The relative ITIH1 mRNA expression in MHCC97H cells and HLF cells treated with YTHDF1 or YTHDF2 siRNAs. C) Results of RNA pulldown with ITIH1‐wt or ITIH1‐M5 in 293T cells. D) Relative ITIH1 levels in MHCC97H‐shMETTL3 cells treated with TGF‐β or galunisertib as determined by RIP with an anti‐YTHDF2 antibody in MHCC97H cells. E) Schematic diagram of YTHDF2‐wt and YTHDF2‐mut. F) The relative YTHDF2 and ITIH1 mRNA levels in MHCC97H cells cotransfected with YTHDF2‐mut and ITIH1‐M5. G) The protein levels of YTHDF2 and ITIH1 in MHCC97H cells cotransfected with YTHDF2‐mut and ITIH1‐M5. Data are represented as means ± SD in the bar graphs. ns: not significant, ^*^: *p* < 0.05, ^**^: *p* < 0.01, ^***^: *p* < 0.001. Gal, galunisertib.

Previous studies suggested that m^6^A modification has been shown to mediate the fate of mRNA. We wondered how m^6^A modification affected *ITIH1* mRNA. After using actinomycin D to block mRNA transcription, the degradation rate of *ITIH1* mRNA decreased when METTL3 was knocked down and increased with overexpression of METTL3. However, TGF‐β stimulation enhanced the degradation of *ITIH1* while galunisertib treatment counteracted this effect (**Figure** **3**A; Figure S5A, Supporting Information), suggesting that TGF‐β regulates the decay of *ITIH1* mRNA mostly in a METTL3‐dependent manner. It is reported that YTHDF2 recruits the mRNA degradation system to decrease mRNA stability.^[^ ^34^, ^35^ ^]^ When YTHDF2 was knocked down, the level of *ITIH1* mRNA increased. YTHDF1, another m^6^A “reader”, had minimal effect on *ITIH1* mRNA (Figure 3B). The RNA pulldown assay indicated that YTHDF2 binds to *ITIH1*‐wt but not *ITIH1*‐M5 mRNA (Figure 3C). To further confirm the association of YTHDF2 and *ITIH1* mRNA, we performed a RIP assay and found that METTL3 knockdown decreased the enrichment of YTHDF2. TGF‐β facilitated YTHDF2 binding, which was abolished by the knockdown of METTL3 and galunisertib treatment (Figure 3D; Figure S5B, Supporting Information). When YTHDF2 was knocked down, the decay rate of *ITIH1* was increased even though under the stimulation of TGF‐β (Figure S5C,D, Supporting Information). This result preliminarily indicated that TGF‐β regulated ITIH1 in a YTHDF2‐dependent manner. Next, we utilized *YTHDF2*‐mut (W432A and W486A), which no longer recognized m^6^A modification (Figure 3E). Western blot and qPCR analyses showed that *YTHDF2*‐wt but not *YTHDF2*‐mut negatively regulated the expression of *ITIH1*‐wt, while *YTHDF2*‐wt had minimal effect on the expression of *ITIH1*‐M5 (Figure 3F,G). GEPIA analysis showed that YTHDF2 expression was negatively correlated with ITIH1 expression and positively correlated with METTL3 expression (Figure S5E, Supporting Information). In addition, the DFS (disease‐free survival) and OS (overall survival) of METTL3 and YTHDF2 exhibited a similar trend (Figure S5F,G, Supporting Information). These results indicated that YTHDF2 recognizes the METTL3‐mediated m^6^A modification of *ITIH1* mRNA and regulates its stability.

### 2.4 ITIH1 Inhibits HCC Development In Vitro and In Vivo

**Figure 4:**
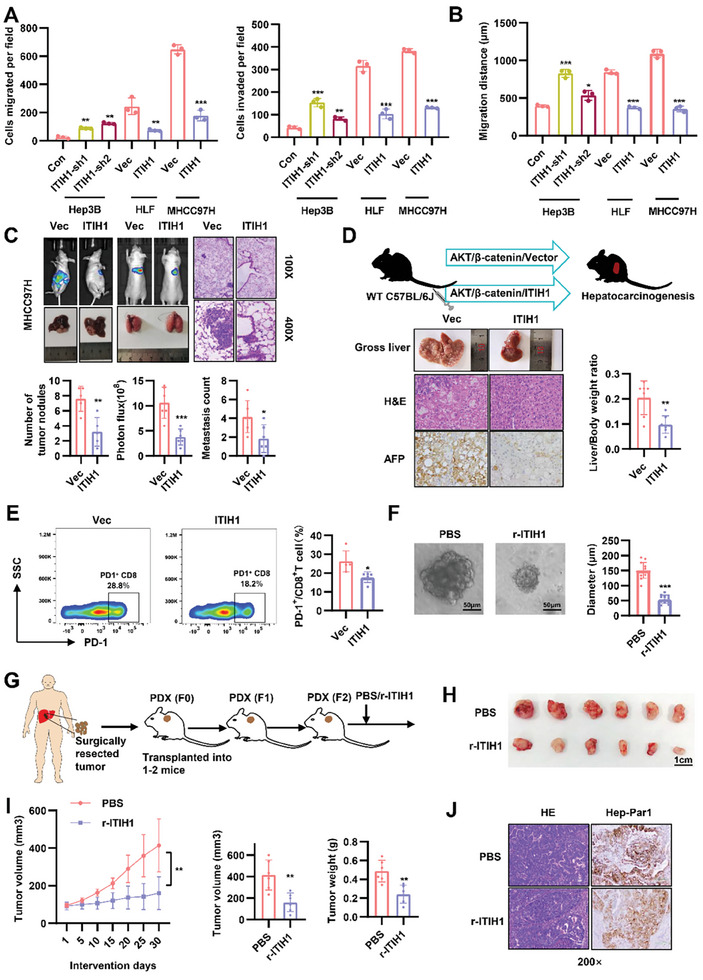
ITIH1 inhibits HCC development in vitro and in vivo. A) The statistical results of the Transwell migration and invasion assays in Hep3B‐shITIH1, HLF‐oeITIH1, and MHCC97H‐oeITIH1 cells. B) The statistical results of the wound healing assay in the indicated stable cell lines. C) The results of the orthotopic transplantation model and tail vein injection model in nude mice established using MHCC97H‐luc‐ITIH1 or vector cells. The statistical results are presented in the lower graphs. Representative image of H&E staining of lung metastases. D) Livers were collected for weighing and IHC staining in HTVi model. E) The proportion of PD‐1^+^ cells in CD8^+^ T cells was detected by flow cytometry. F) Representative images of HCC organoids treated with PBS or r‐ITIH1 for 10 days and quantification of organoid diameters. G) Schematic diagram of the generation of the PDX model. Patient‐derived tumor materials were xenografted and passed in NCG mice. H) PDX models in NCG mice treated with or without r‐ITIH1. Twenty‐five days after intervention, the tumor volume and tumor weight in the livers were determined. I) After each intervention, the tumor volume and tumor weights were calculated. J) H&E and hepatocyte staining were performed in the tumors. Data are represented as means ± SD in the bar graphs. ns: not significant, ^*^: *p* < 0.05, ^**^: *p* < 0.01, ^***^: *p* < 0.001.

To explore the function of ITIH1 in HCC, we tested its expression level in different cell lines (Figure S6A, Supporting Information). In Hep3B cells which express high levels of ITIH1, shRNAs‐mediated knockdown of ITIH1 led to increased cell migration and invasion. On the contrary, overexpression of ITIH1 in HLF and MHCC97H suppressed cell motility (**Figure** **4Al**; Figure S6B,C, Supporting Information). Similar effects were observed in the wound healing assay (Figure 4B; Figure S6D, Supporting Information).

To evaluate the tumor‐suppressive role of ITIH1 in HCC in vivo, we applied various xenograft models. First, we utilized the orthotopic model to examine intrahepatic metastasis of HCC cells 5 weeks post‐inoculation. Injection of ITIH1‐overexpressing cells substantially decreased the capacity of HCC cells to form secondary lesions in the liver (Figure 4C; Figure S6E, Supporting Information). In the lung metastasis model, control or ITIH1‐overexpressed cells were injected through the tail vein of the mice. Six weeks after injection, fewer lesions were found in mice injected with ITIH1‐overexpressed cells (Figure 4C; Figure S6E, Supporting Information). To evaluate the effect of ITIH1 on the development of HCC, we used hydrodynamic tail vein injection (HTVi) with AKT/β‐catenin plasmids. Mice developed HCC 16 weeks after injection. IHC staining showed weakened AFP expression in the ITIH1‐overexpressed group and the liver/body weight ratio in this group was lower than that in the vector group (Figure 4D). Furthermore, in this model, we analyzed the immune cells infiltrating the tumors in mice. There were no differences in the populations of macrophages, myeloid‐derived suppressor cells (MDSCs), B cells, NK cells, and T cells in the tumor microenvironment between the two groups (Figure S7A, Supporting Information). Considering the crucial role of T cells in tumor killing, we further conducted flow cytometry analysis on T cell subsets and found that CD8^+^ T cells in the T cell population had a higher abundance in the tumors after overexpressing ITIH1 (Figure S7B, Supporting Information). Additionally, the proportion of PD‐1^+^CD8^+^ T cells decreased after ITIH1 overexpression, suggesting a reduction in CD8^+^ T cell exhaustion (Figure 4E). These results indicated that ITIH1 inhibited the development of HCC. Since ITIH1 is a secreted protein located in the extracellular matrix, we then used purified ITIH1 (recombinant ITIH1, r‐ITIH1) to evaluate the tumor suppressive effect in vivo (Figure S7C, Supporting Information). To investigate whether the r‐ITIH1 protein has a therapeutic effect on HCC, we established an organoid model from an HCC patient. It was found that r‐ITIH1 could significantly inhibit the growth of HCC organoids (Figure 4F). In another HCC patient‐derived xenograft (PDX) model (Figure 4G), r‐ITIH1 was injected near the tumor. Of note, r‐ITIH1 significantly suppressed the growth of PDX tumors (Figure 4H–J). In summary, these data indicated that ITIH1 acts as a tumor suppressor during HCC progression.

### 2.5 ITIH1 Interacts with Integrin α5β1

**Figure 5:**
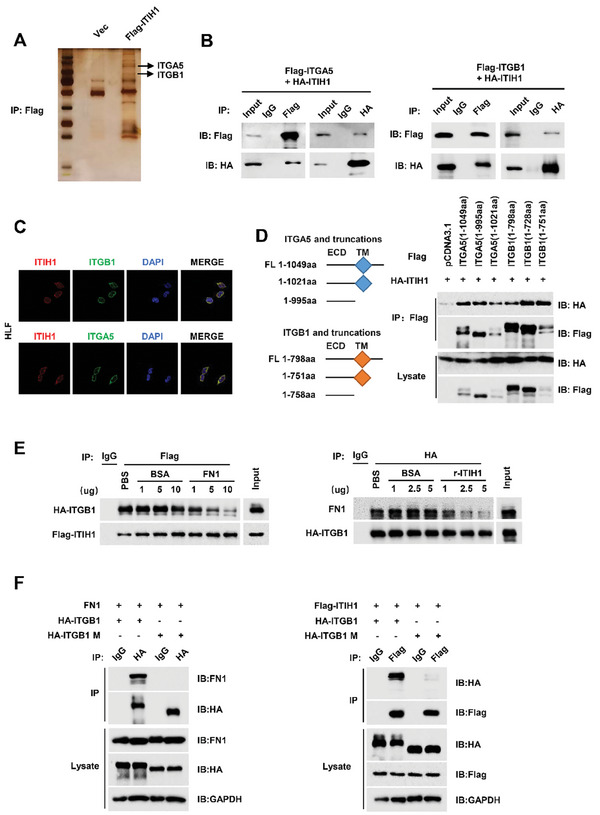
ITIH1 interacts with integrin α5β1. A) The results of silver staining after IP. B) The results of IP with HA‐ITIH1+Flag‐ITGA5 and HA‐ITIH1+Flag‐ITGB1 in 293T cells. C) Confocal images of IF staining for ITIH1+ITGB1 and ITIH1+ITGA5 in HLF cells. D) Schematic diagram of the ITGA5 and ITGB1 truncation mutant constructs. Results of IP with ITIH1 and the ITGB1 and ITGA5 truncation mutant constructs in 293T cells. E) The results of IP with ITIH1+ITGB1 and ITGB1+FN1 when FN1 or r‐ITIH1 were added in a concentration gradient in 293T cells. F) The results of IP with ITGB1‐wt or deletion mutants of ITGB1 and FN1 or ITIH1 in 293T cells. Data are represented as means ± SD in the bar graphs. ns: not significant, ^*^: *p* < 0.05, ^**^: *p* < 0.01, ^***^: *p* < 0.001.

We next explored the potential mechanism of ITIH1‐induced restraint of HCC metastasis and development. Immunoprecipitation (IP) followed by mass spectrometry (MS) was utilized to identify potential ITIH1 interaction partners (**Figure** **5**A). The integrin family proteins were verified and analyzed (Figure S8A, Supporting Information). As a functional complex, integrins are made up of α and β subunits. ITGB1 and ITGA5 are paired subunits that transduce extracellular signals into cells. The co‐IP result showed that ITIH1 had higher binding affinity to ITGB1 and ITGA5 in vitro and in vivo compared with other integrin family proteins (Figure 5B; Figure S8B,C, Supporting Information). Immunostaining demonstrated that ITIH1 is associated with ITGB1 and ITGA5 mostly in the cell membrane and partly in the cytoplasm (Figure 5C; Figure S8E, Supporting Information). Further analysis showed that ITIH1 interacted with the extracellular domains of ITGB1 and ITGA5 (Figure 5D).

It has been reported that FN (Fibronectin) is an important ligand of the ITGB1/ITGA5 complex and that its binding leads to activation of the integrin/FAK signaling pathway.^[^ ^31^ ^]^ We then confirmed that ITIH1 directly binds to ITGA5/ITGB1 and besides, ITIH1/FN competitively binds to ITGB1 in a dose‐dependent manner (Figure 5E; Figure S8D, Supporting Information). However, ITIH1 did not affect the ITGA5/FN complex (Figure S8F, Supporting Information). To further verify this observation, we generated an ITGB1 mutant (ITGB1 M) with the deletion of amino acids 130–240, which exhibits an impaired ability to bind to FN but is still localized on the cell surface. The ITGB1 mutant did not interact with FN or ITIH1, indicating that FN and ITIH1 interact with ITGB1 via the same protein domain (Figure 5F). Together with the above evidence, ITIH1 acts as a new ligand for ITGB1/ITGA5 and competes with FN for its binding to ITGB1.

### 2.6 ITIH1 Combined with Galunisertib has Potential Therapeutic Effects on HCC Progression

**Figure 6:**
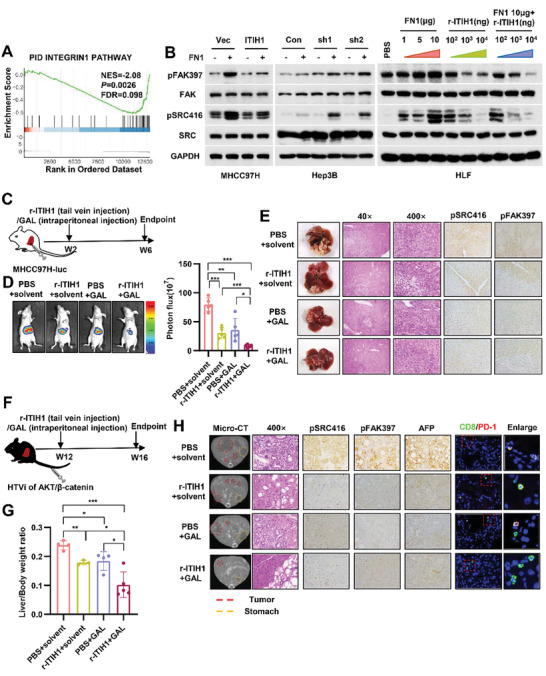
ITIH1 inhibits the integrin/FAK signaling pathway. A) Gene set enrichment analysis showed that integrin beta1 signaling pathway‐related genes in the PID database were significantly enriched in control HLF cells compared with HLF cells with ITIH1 overexpression. B) MHCC97H‐oeITIH1 and Hep3B‐shITIH1 cells were treated with FN1 stimulation for 2 h. Then, the cells were collected for WB analysis. HLF cells were treated with FN1 and r‐ITIH1 alone or in combination in a concentration gradient. Then, the cells were collected for WB analysis 2 h after stimulation. C) MHCC97H‐luc cells were orthotopically injected into the livers of nude mice. Two weeks after injection, the mice received galunisertib intraperitoneally and r‐ITIH1 via the tail vein alone or in combination. Four weeks later, luciferase activity was measured D). E) The livers of nude mice were harvested and subjected to H&E and IHC staining. F–H) The indicated plasmids were injected into C57 mice via the tail vein, and 12 weeks after injection, the mice were treated as described above. Four weeks later, the mice were subjected to micro‐CT scanning, After the livers of C57 mice were weighed, the liver weight/body weight ratios were calculated (G), and their livers were then collected for IHC staining (H). Data are represented as means ± SD in the bar graphs. ns: not significant, ^*^: *p* < 0.05, ^**^: *p* < 0.01, ^***^: *p* < 0.001. Gal, galunisertib.

We next tested whether ITIH1 has an effect on downstream signaling in integrin pathways. RNA‐sequencing was performed in ITIH1‐overexpressed and control HLF cells (Figure S9A, Supporting Information). Kyoto Encyclopedia of Genes and Genomes (KEGG) pathway enrichment analysis indicated that genes regulated by ITIH1 overexpression exhibited the greatest enrichment in the focal adhesion pathway (Figure S9B, Supporting Information). Gene set enrichment analysis (GSEA) results indicated that genes induced by integrin/FAK signaling were significantly enriched in control cells compared with ITIH1‐overexpressed cells (**Figure** **6**A; Figure S9C, Supporting Information). Collectively, these results indicated that ITIH1 expression is negatively correlated with integrin/FAK activation and might inhibit this pathway.

To experimentally verify this hypothesis, HCC cells were treated with ITIH1 depletion or overexpression with or without FN for stimulation (Figure 6B; Figure S10A, Supporting Information). The results showed that ITIH1 overexpression reduced and ITIH1 depletion increased FN‐dependent activation of FAK signaling, as indicated by FAK phosphorylation at Tyr397 and SRC phosphorylation at Tyr416; in addition, TGF‐β stimulation enhanced FN‐dependent activation of FAK signaling, and galunisertib treatment abolished the effect of TGF‐β (Figure S10B, Supporting Information). Likewise, r‐ITIH1 treatment significantly reduced the activation of FAK signaling accompanying FN stimulation in a dose‐dependent manner (Figure 6B). This result also confirmed the biological activity of r‐ITIH1.

As r‐ITIH1 intervention had an effect in the HCC PDX model and galunisertib has already been used in clinical HCC treatment, we hypothesized that r‐ITIH1 might synergize with galunisertib to suppress HCC development in vivo. First, we orthotopically injected luciferase‐expressing MHCC97H cells into immunocompromised mice (Figure 6C). Two weeks after injection, the mice were randomly divided into four groups for administration of the different treatments. Four weeks after treatment, the nude mice were sacrificed, and H&E staining and imaging showed that the tumor volume in the galunisertib+r‐ITIH1 group decreased more significantly than that in the other groups. Also, the inhibitory effect of each treatment on integrin/FAK signaling was verified by IHC staining (Figure 6D,E). To further verify the effect of r‐ITIH1, we established a model of HCC driven by activated AKT and β‐catenin in immunocompetent mice through hydrodynamic tail vein injection (Figure 6F). Twelve weeks after injection, these mice were randomly divided into 4 groups and subjected to various treatments three times per week. In the last week (week 16), they were subjected to micro‐CT scanning (Figure 6H). Before sacrifice, blood biochemical analysis was conducted, and the results suggested that these treatments appeared to be well tolerated and did not cause obvious hepatotoxicity in the mice (Figure S10C, Supporting Information). Both galunisertib and r‐ITIH1 can reduce the liver weight ratio (Figure 6G). Consistent with the results in nude mice, integrin/FAK signaling was significantly inhibited in each treatment group (Figure 6H). Additionally, CD8^+^ T cells were more abundant overall after the combination treatment, whereas the proportion of PD‐1^+^CD8^+^ T cells was lower, indicating a reduction in CD8^+^ T cell exhaustion after the drug combination treatment (Figure 6H). These results indicated that r‐ITIH1 might synergize with galunisertib to suppress HCC development.

### 2.7 ITIH1 Expression is Negatively Correlated with FAK Activation in HCC Patients

**Figure 7:**
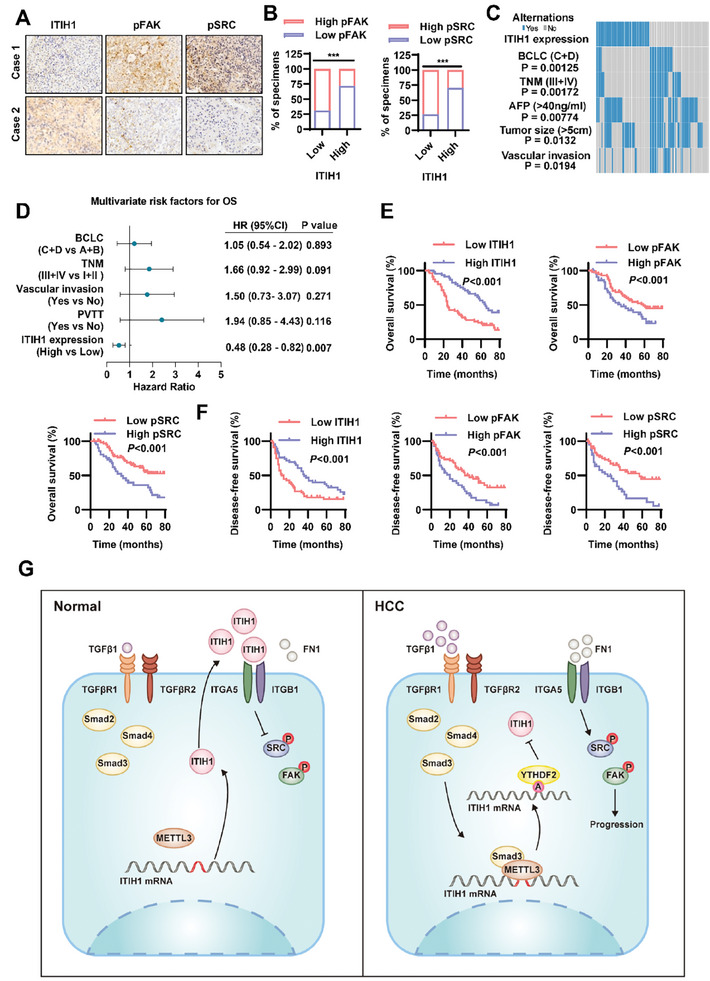
ITIH1 expression is negatively correlated with FAK activation in HCC patients. A) Representative IHC staining results in paraffin‐embedded HCC samples from Tongji Hospital. B) The correlations of pFAK/ITIH1 and pSRC/ITIH1 levels as determined by IHC staining. C) Waterfall plot showing clinicopathological features that were significantly correlated with ITIH1 expression in HCC samples from Tongji Hospital. D) Multivariate regression analysis with the clinicopathological features identified as significantly connected with prognosis in univariate regression analysis in HCC samples from Tongji Hospital. E) The OS prognosis of patients according to ITIH1, pFAK, and pSRC protein levels. F) DFS prognosis of patients according to ITIH1, pFAK, and pSRC protein levels. G) Schematic illustration showing the roles of ITIH1 in HCC cells. Data are represented as means ± SD in the bar graphs. ns: not significant, ^*^: *p* < 0.05, ^**^: *p* < 0.01, ^***^: *p* < 0.001.

IHC analysis revealed that the expression of ITIH1 was negatively correlated with the activation status of FAK and SRC in HCC samples (**Figure** **7**A,B). To further confirm this correlation, we tested the related protein levels in tumor tissues and adjacent nontumor tissues through WB analysis (Figure S11A, Supporting Information). We found that the ITIH1 protein level was decreased in tumor tissues, while the levels of pFAK (Tyr397) and pSRC (Tyr416) were increased in tumor tissues (Figure S11B–D, Supporting Information). Collectively, the WB analysis results indicated that in HCC tissues, the ITIH1 protein level was negatively correlated with the pFAK and pSRC levels (Figure S11E, F, Supporting Information). When these findings were combined with the specific data of HCC patients, low ITIH1 expression was found to be correlated with advanced BCLC stage (*P *= 0.00125), advanced TNM stages (*P *= 0.00172), high AFP expression (*P *= 0.00774), large tumor size (*P *= 0,0132), and increased vascular invasion (*P *= 0.0194, Figure 7C). Univariate regression analysis was conducted on clinicopathological features, including ITIH1 expression, in the cohort of HCC patients from Tongji Hospital. The results showed that BCLC stage, TNM stage, vascular invasion, portal vein tumor thrombus (PVTT), and ITIH1 expression were correlated with overall survival (OS). Multivariate regression analysis of the above factors, we found that ITIH1 expression was an independent factor associated with overall survival (Figure 7D). Consistent with this finding, a high ITIH1 protein level, low pFAK (Tyr397) level, and low pSRC (Tyr416) level were correlated with better DFS (disease‐free survival) and OS (Figure 7E,F).

Ultimately, our research discovered that ITIH1, whose expression was regulated by both TGF‐β stimulation and m^6^A modification, inhibited HCC metastasis by inhibiting the integrin/FAK signaling pathway, and we preliminarily verified the effectiveness of the application of recombinant ITIH1 to suppress HCC metastasis (Figure 7G).

## 3 Discussion

During cancer development, the TGF‐β signaling pathway plays crucial roles in the different stages of cancer^[^ ^10^ ^]^; m^6^A mRNA modification also affects tumor progression through various mechanisms, including liquid–liquid phase separation.^[^ ^36^ ^]^ SMAD2/3 promotes the binding of the m^6^A methyltransferase complex to a subset of transcripts involved in early cell fate decisions.^[^ ^15^ ^]^ However, little is known regarding the relationship between TGF‐β and m^6^A modification in HCC development. First, we confirmed their interaction in HCC cells. SMAD3 promotes the liquid–liquid phase separation of METTL3. Then, we verified the role of an ECM protein, ITIH1, which is regulated by TGF‐β stimulation but independent of the transcriptional function of TGF‐β. In this study, ITIH1 was found to be the direct downstream target of TGF‐β signaling and m^6^A modification. Consistent with this finding, when METTL3‐mut was expressed instead of METTL3‐wt, TGF‐β stimulation did not lead to the downregulation of ITIH1. Interestingly, according to the results that we have obtained, the knockdown of METTL3 could not impede the effect of the TGF‐β‐SMAD3 signaling pathway on ITIH1. We hypothesized that SMAD3 functioned in facilitating *ITIH1* RNA m^6^A modification requiring the RNA‐binding activity of SMAD3. Next, when m^6^A‐modified ITIH1 mRNA is translocated into the cytoplasm, it is recognized by a reader, YTHDF2, and then degraded. This process might explain why we detected lower ITIH1 expression in HCC tumor tissues. The specific mechanisms by which TGF‐β promotes LLPS (Liquid‐Liquid Phase Separation) of METTL3 through SMAD3 remain to be further explored.

During cancer progression, ECM proteins always exert promoting effects.^[^ ^37^, ^38^ ^]^ However, the ECM components with inhibitory effects remain to be explored. In our study, we found that when ITIH1 is secreted from cells, it can compete with FN for binding to the ITGB1/ITGA5 complex and then inhibit the activation of integrin/FAK signaling. In line with our finding that high ITIH1 expression correlates with good clinical outcomes in HCC patients, both ITIH1 overexpression and recombinant ITIH1 treatment inhibited tumor metastasis and development without causing significant toxicity in mice, indicating the potential therapeutic effects of ITIH1.

Integrins are crucial surface receptors that connect intracellular structures and ECM components and transduce important signals. In tumor cells, these paired integrins interact with FN and other ECM proteins to facilitate cancer progression.^[^ ^39^, ^40^ ^]^ In our study, we confirmed the binding between ITIH1 and the extracellular domains of ITGB1 and ITGA5. Integrins are functional complexes composed of α and β subunits. In contrast to the hypothesized association, ITIH1, as a ligand for the ITGB1/ITGA5 complex, competes with FN for binding to ITGB1 rather than to ITGA5. The underlying mechanisms of these three molecules need to be further explored.

Galunisertib, a new TGFβRI inhibitor, has been used alone and in combination with other treatments in the clinic.^[^ ^14^ ^]^ In a subset of patients with advanced HCC treated with galunisertib, decreases in the circulating AFP and TGF‐β1 levels were connected with longer OS times.^[^ ^11^ ^]^ In our study, we verified the therapeutic effect of galunisertib monotherapy in HCC in different mouse models. In addition, we added a new group treated with galunisertib+r‐ITIH1, and the mice in this group had the lowest liver/body weight ratios. This result indicates that the resulting synergism produced a better inhibitory effect on HCC development in our mouse models. In one of our mouse models, we delivered myr‐AKT and δN90‐β‐catenin by hydrodynamic tail vein injection.^[^ ^41^ ^]^ After anatomical examination, the tumors in the livers of mice metastasized throughout the body, and it was difficult to count the tumors and calculate the volume of each tumor. Moreover, there was no obvious difference in AFP, p‐FAK, and p‐Src staining among the three treatment groups. Differences were observed only between the vector group and the treatment groups. In the CDX (cell‐derived xenograft) model in nude mice, the difference in tumor growth among the three treatment groups was more obvious. To further verify the synergistic effect of galunisertib and r‐ITIH1, more animal experiments need to be performed.

More interestingly, ITIH1 is a protein that is only expressed in the liver, according to NCBI RNA‐seq data. In addition to its treatment potential, it might also be a diagnostic biomarker for HCC.^[^ ^42^ ^]^ Future studies should explore the potential diagnostic value of ITIH1 in HCC and establish a critical level, similar to AFP, to diagnose HCC at an early stage.

## 4 Experimental Section

### Patient Samples

Human liver tissue samples were collected from patients who received surgical resection between 2010 and 2014 at the Hepatic Surgery Centre, Tongji Hospital of Huazhong University of Science and Technology (Wuhan, China). A set of 76 pairs of snap‐frozen HCC tissues and adjacent nontumor liver tissues were used for Western blot analysis. Ethical approval was obtained from the Ethics Committee of Tongji Hospital of HUST.

### Animal Studies

All procedures involving mice and the corresponding experimental protocols were performed under the guidelines for the care and use of laboratory animals and were approved by the Ethics Committee of Tongji Hospital of Huazhong University of Science and Technology. The different models are described as follows: i) The orthotopic transplantation model was used to study in vivo metastasis within the liver. Briefly, HCC cells stably transfected with a firefly luciferase expression vector (1 × 10^6^ cells 30 µL^−1^) were injected into the left liver lobe of 5‐week‐old male BALB/c nude mice. Luciferase bioluminescence in each mouse was measured at week 6 after injection. Mice in all the groups were sacrificed 8 weeks after injection. ii) For the lung metastasis model, we slowly injected HCC cells (1 × 10^6^ cells 100 µL^−1^) into 5‐week‐old male BALB/c nude mice via the tail vein. Six weeks after injection, luciferase bioluminescence was measured, and the mice were then sacrificed. iii) As described in the previous study, 6‐8‐week‐old male wild‐type C57BL/6J mice were used for hydrodynamic tail vein injection (HTVi). The pT3‐EF1α‐myr‐AKT‐HA (human), pT3‐EF1α‐△N90‐β‐catenin (human), and pT3‐EF1α‐ITIH1 (human) plasmids were constructed by using the pT3‐EF1α vector. The pooled pT3 plasmids and the Sleeping Beauty transposon plasmid (pCMV‐SB) were mixed at a ratio of 25:1 and 20 µg of each pT3 plasmid was used for each mouse. The plasmid mixture was then diluted in sterile PBS, and a total volume of 2 mL per mouse was finally injected into the tail vein of mice over a period of 5–7 s. Expression of the target gene is maintained in the liver over a long period of time through this rapid hydrodynamic injection method. iv) For the human patient‐derived xenograft (PDX) model, the standard protocol for PDX transplantation, maintenance, and tumor digestion was followed. NCG mice(4‐5 weeks old, strain NO.T001475)were bought from GemPharmatech (Nanjing, China). The tumor was cut into small pieces (3×3×3 mm^3^), inoculated subcutaneously into mice, waited it to grow to become F0 generation, and continued to pass through to other mice to allow the tumor to grow stably. For treatments, mice of F2 generation were randomly divided into 2 groups. Each group was treated with recombinant ITIH1 (r‐ITIH1, 20 µg in 50 µL PBS, once every 2 days) or 50 µL PBS. v) For combination treatment of r‐ITIH1 and galunisertib, mice were injected with 20 µg r‐ITIH1/mouse three times per week via the tail vein; for galunisertib treatment, mice were intraperitoneally injected with 50 mg k^−1^g galunisertib three times per week.

### Preparation of ITIH1‐Fc Recombinant Protein

The cDNA sequence encoding the ITIH1 protein was PCR amplified and subcloned into the pINFUSE‐hIgG2‐Fc2 vector (InvivoGen, cat. no. pfc2‐hgin2) with a human IgG2Fc tag. The ITIH1‐Fc expression vector was transiently transfected into 293T cells by using FectoPRO transfection reagent from PolyPlus according to the manufacturer's instructions (cat. no. 116‐040). Two to three days after transfection, cell supernatants were harvested and purified by affinity chromatography with protein A under the manufacturer's purification system protocol. SDS–PAGE and Coomassie blue staining were used to analyze the protein preparation, and only one major band at the predicted molecular weight was observed.

### HCC Organoid Culture

Fresh tumor tissue from HCC patients was collected, soaked, and washed five times in a DMEM medium containing Penicillin–Streptomycin. It was then minced in a sterile environment and resuspended in DMEM containing DNase I (0.1 mg mL^−1^), Collagenase IV (2.5 mg mL^−1^), and Dispase II (2 mg mL^−1^, Roche, 0,494,207,8001). Furthermore, it was digested by shaking at 37 °C for 30 min. The suspension was filtered through a 70 µm cell strainer, and red blood cells were removed using red blood cell lysis buffer. The cells were washed with cold PBS, centrifuged at 400 g for 5 min, and resuspended in cold basement membrane extract (Cultrex Reduced Growth Factor Basement Membrane Extract, Type 2, Pathclear, R&D Systems, 3533‐010‐02). The amount of basement membrane extract used was adjusted based on the tissue amount and cell density. Finally, 50 µL of cell‐containing basement membrane extract was added to a preheated 24‐well plate at 37 °C, and the plate was placed in a 37 °C incubator. After 30 min, the basement membrane extract solidified, and 600 µL of organoid culture medium (Advanced DMEM/F12 containing 1% Penicillin–Streptomycin, 1% GlutaMAX, 1% HEPES, 2% B27, 1% N‐2, 1.25 mm, 10 mm Nicotinamide, 100 ng mL^−1^ WNT3a, 50 ng mL^−1^ EGF, 100 ng mL^−1^ FGF10, 25 ng mL^−1^ HGF, 25 ng mL^−1^ Noggin, 100 ng mL^−1^ R‐spondin‐1, 10 µm Y‐27632, 5 µm A83‐01, 10 µm Forskolin) was added. After 3 days, the medium was replaced with the organoid culture medium mentioned above, without Y‐27632, for continued cultivation.

### Statistical Analysis

Statistical analysis was performed using SPSS 13.0 (SPSS, Chicago, IL, USA) or GraphPad Prism 5.0 (GraphPad, La Jolla, CA, USA) software. All values are expressed as the means ± SEMs. Quantitative data were analyzed by two‐tailed Student's *t*‐test, analysis of variance (ANOVA) with the Bonferroni post hoc test, or Pearson correlation analysis when applicable or with a nonparametric test such as the Wilcoxon signed‐rank test, Mann–Whitney U test or Spearman rank correlation analysis. Categorical data were analyzed by the χ2 test. The Kaplan–Meier method with the log‐rank test was used to assess differences in survival between subgroups. Significant differences between the two groups are denoted by asterisks (^*^ *p* < 0.05; ^**^ *p* < 0.01; ^***^ *p* < 0.001).

## Conflict of Interest

The authors declare no conflict of interest.

## Author Contributions

Z.L., H.Z., F.L., and W.W. contributed equally to this work. H.W.Z., Z.L., B.Z., and X.C. have designed and conducted the experiments. H.W.Z., Z.L., F.L., and W.W. have performed the experiments. H.W.Z., Y.L., C.S., L.Z., and H.Z. have given the technical or material support. H.W.Z., F.L., and W.W. have done the data analysis. H.W.Z., Z.L., Z.Z., and B.Z. have written and edited the manuscript. Z.L., B.Z., Z.Z., and X.C. have obtained the funding and supervised the study. All authors have access to the study data and have reviewed and approved the final manuscript.

## Supporting information

Supporting Information

## Data Availability

The data that support the findings of this study are available from the corresponding author upon reasonable request.
