# Supplementary material for: m6A‐Dependent ITIH1 Regulated by TGF‐β Acts as a Target for Hepatocellular Carcinoma Progression
Source: Adv Sci (Weinh). 2024 Sep 5;11(42):2401013. doi: 10.1002/advs.202401013 (PMC11558142; doi:10.1002/advs.202401013)
Supplement: Supplementary file 1 — Supporting Information [file ADVS-11-2401013-s001.docx]

**Supplementary Materials**

**m6A-dependent ITIH1 regulated by TGF-β acts as a target for hepatocellular carcinoma progression**

Zhibin Liao^1,2§^, Hongwei Zhang^1,2§^, Furong Liu^1,2§^, Weijian Wang^1,2§^, Yachong Liu^1,2^, Chen Su^1,2^, He Zhu^1,2^, Xiaoping Chen^1,2,3*^, Bixiang Zhang^1,2, 3*^, Zhanguo Zhang^1,2*^

**Antibodies and reagents**

Mouse serum IgG (I5381 for IP control), rabbit serum IgG (I5006 for IP control), and primary antibodies against Flag (F1804 for WB, IP and IF analyses) and HA (H6908 for WB, IP and IF analyses) were obtained from Sigma (St. Louis, MO, USA); the primary antibody against AFP (ab284388) was purchased from Abcam (Cambridge, MA, USA); primary antibodies against phospho-FAK (Tyr397) (#3283S), FAK (#3285S), phospho-Src (Tyr416) (#6943S), Src (#2109S), METTL3 (#86132S), SMAD2 (#5339S), SMAD3 (#9523S), pSMAD2-Ser465/467 (#18338 for WB analysis), and YTHDF2 (#71283S) were obtained from CST (Danvers, MA, USA); the primary antibody against pSMAD3 (Ser423/425) (#07-1389 for WB analysis) was obtained from Millipore (Darmstadt, Germany); primary antibodies against GAPDH (#60004-1-Ig), Fibronectin (#15613-1-AP), ITGB1 (#12594-1-AP), and ITGA5 (#10569-1-AP) were purchased from Proteintech (Wuhan, Hubei, China); the primary antibody against ITIH1 (HPA042049) was obtained from Atlas Antibodies; and HRP-conjugated anti-rabbit IgG (#111-035-003 for WB analysis) and HRP-conjugated anti-mouse IgG (#115-035-003 for WB analysis) were obtained from Jackson ImmunoResearch Laboratories (PA, USA). HRP-conjugated anti-rabbit IgG light chain (A25022 for WB analysis), HRP-conjugated anti-mouse IgG light chain (A25012 for WB analysis), FITC-conjugated goat anti-mouse IgG (A22110 for IF analysis), DyLight549-conjugated goat anti-rabbit IgG (A23320 for IF analysis), DyLight549-conjugated goat anti-mouse IgG (A23310 for IF analysis) and DyLight649-conjugated goat anti-rabbit IgG (A23620 for IF analysis) were purchased from Abbkine (California, USA). All inhibitors were purchased from Selleck (Houston, TX, USA).

**Plasmids and constructions**

A 1.0 kb fragment of the ITIH1 promoter sequence was cloned into the pGL4.17 vector. The ITIH1-wt and ITIH1-mut5 mRNA sequences were inserted into the psiCHECK2 vector. Mammalian expression plasmids for the Flag- and HA-tagged CDSs were constructed by a standard molecular cloning method from cDNA templates. The point mutant plasmids and truncation mutant plasmids were constructed by site-directed mutagenesis. All constructs were confirmed by DNA sequencing.

**Lentivirus-transduced stable cell lines**

To generate cell lines with gene overexpression or knockdown, the Flag-tagged CDS of ITIH1 was inserted into the BamHI/SalI sites in the pLenti-CMV-Puro plasmid (Addgene #17448), and the short hairpin RNA sequences targeting ITIH1 were inserted into the pLKO.1-TRC vector (Addgene #10879). Vectors containing a Flag tag or scrambled shRNA sequence (Addgene #1864) were used as the corresponding negative controls. Lentivirus was produced in HEK293 cells by cotransfection of pMD2.G (Addgene #12259), psPAX2 (Addgene #12260) and the pLenti-CMV-Puro or pLKO.1-shRNA plasmid (ratio 1:3:4). Virus-containing supernatant was collected 48 hours post-transfection and passed through 0.45 µm filters prior to PEG-8000 precipitation. Aliquots were stored at -80 °C. To generate stable cell lines, lentiviral stocks were used to transduce MHCC97H, HLF or Hep3B cells in the presence of 8 µg/ml polybrene. The target sequences for ITIH1 were as follows: shITIH1-1, 5’-CCGGGCAGTATGAAATTGTCATCAATTGATGACAATTTCATACTGCTTTTTG-3’; shITIH1-2, 5’-CCGGCCATGCCTCAATACTCATCATATGATGAGTATTGAGGCATGGTTTTTG-3’.

**Quantitative reverse transcription PCR**

FastPure®Cell/Tissue Total RNA Isolation Kit V2（RC112,Vazyme Biotech Co.,Ltd） was used according to the manufacturer’s protocol to extract total RNA from cells. Reverse transcription of mRNA was performed using a reverse transcription system kit (Takara, Otsu, Japan). qPCR analysis was performed with a standard SYBR Green PCR Kit (Toyobo Life Science, Osaka, Japan) according to the manufacturer’s protocols. GAPDH was used as the endogenous control for the determination of mRNA expression levels. Relative quantification was performed using the comparative CT (2^-ΔΔCT^) method. Each assay was repeated three times independently. The primers used in this study were as follows: GAPDH-F, 5’-GACAAGCTTCCCGTTCTCAG-3’ and GAPDH-R, 5’-GAGTCAACGGATTTGGTCGT-3’; ITIH1-F, 5’-GAGGCACTCCTTAAAATTCTGGG-3’ and ITIH1-R, 5’- GCCCTTCCACGATTGTACTCG-3’; GDF15-F, 5’- GACCCTCAGAGTTGCACTCC-3’ and GDF15-R, 5’- GCCTGGTTAGCAGGTCCTC-3’; TGM2-F, 5’- GAGGAGCTGGTCTTAGAGAGG-3’ and TGM2-R, 5’- CGGTCACGACACTGAAGGTG-3’; EGFR-F, 5;- AGGCACGAGTAACAAGCTCAC-3’ and EGFR-R, 5’- ATGAGGACATAACCAGCCACC-3’; MMP24-F, 5’- GCCGGGCAGAACTGGTTAAA-3’ and MMP24-R, 5’- CCCGTAAAACTGCTGCATAGT-3’; HMOX1-F, 5’- AAGACTGCGTTCCTGCTCAAC-3’ and HMOX1-R, 5’- AAAGCCCTACAGCAACTGTCG-3’; DUSP6-F, 5’- GAAATGGCGATCAGCAAGACG-3’ and DUSP6-R, 5’- CGACGACTCGTATAGCTCCTG-3’; SOCS2-F, 5’- TTAAAAGAGGCACCAGAAGGAAC-3’ and SOCS2-R, 5’- AGTCGATCAGATGAACCACACT-3’; JUN-F, 5’- TCCAAGTGCCGAAAAAGGAAG-3’ and JUN-R, 5’- CGAGTTCTGAGCTTTCAAGGT-3’; SETBP1-F, 5’- CCAACGCGGACAGTGAGAAAT-3’ and SETBP1-R, 5’- CCTCCTTCGTGGCTTTGCTAT-3’; RRP12-F, 5’- GTGACCTGACAGTCGATGCTG-3’ and RRP12-R, 5’- GTGACGTTTGTGCAGTCGG-3’; FOXD2-F, 5’-TTGGGTGGAAGAGAAGGGTC-3’ and FOXD2-R, 5’- TTTCACAGAGCTTCCCCACA-3’.

**Transwell cell migration and invasion assays**

Cell migration and invasion assays were performed in 24-well Transwell plates (8 μm pore size, Corning, NY, USA) according to the manufacturer's instructions. For the cell invasion assay, the filter membranes were precoated with 50μl of a 1:4 mixture of Matrigel (BD Biosciences, NJ, USA) and DMEM for 4 hours at room temperature. Culture medium containing 10% FBS was added to the lower compartments, and cells (HLF and MHCC97H, 2×10^4^; Hep3B, 5×10^4^) in 100 μl of FBS-free medium were seeded into the upper compartments. After 24 hours of culture, nonmigrated or noninvaded cells were removed by scraping the upper surface of each membrane with a cotton swab. Cells on the lower surface of each filter membrane were fixed with paraformaldehyde and stained with crystal violet. Cells were counted under an optical microscope. Each experiment was repeated at least three times.

**Wound healing assay**

A confluent monolayer of HCC cells (HLF, MHCC97H, and Hep3B) was generated by overnight culture, and a scratch was made with a 10 µl pipette tip. Cell migration was recorded by phase contrast microscopy (Nikon Digital ECLIPSE C1 system, Nikon Corporation) at the indicated time points. Images of 6 random fields were acquired for quantitative analysis. Each experiment was repeated at least three times.

**Immunoprecipitation**

Cells were collected and lysed in IP lysis buffer. Supernatants were collected by centrifugation (15,000 × g, 15 min, 4 °C) and were precleared with 10 µl of Ig-A/G-magnetic beads (BioLinkedIn, China) prior to centrifugation at 1,500 × g for 2 min at 4 °C. The precleared supernatants were incubated with the indicated antibodies (1 µg/ml) overnight at 4 °C and then subjected to immunoprecipitation with 20 µl of protein G-conjugated agarose for 2 hours at 4 °C. The precipitates were washed 5-7 times with IP wash buffer, and immunocomplexes were detected by WB analysis. For endogenous co-IP experiments, precipitates were washed 3 times instead of 5-7 times with IP lysis buffer, and immunocomplexes were detected by WB analysis. For the specific reagents used, we referred to this study.

**Immunohistochemical (IHC) staining and scoring of ITIH1, p-FAK, and p-Src IHC staining levels in HCC samples**

Immunohistochemical staining of tissues was performed by using a polymer HRP detection system (Zhongshan Goldenbridge Biotechnology) on paraffin-embedded HCC tissue sections. The paraffin sections were dewaxed, antigen retrieval was performed with 0.01 M sodium citrate buffer (pH 6.0), and the sections were incubated with 3% hydrogen peroxide for 20 min at room temperature to block endogenous peroxidase activity prior to blocking with 5% bovine serum albumin for 60 min. The slides were incubated overnight at 4 °C with antibodies against ITIH1, p-FAK, and p-Src in a humidified chamber and were then incubated with an HRP-conjugated secondary antibody for 60 minutes at room temperature. Antibody binding was detected with DAB, and the reaction was stopped by immersing the tissue sections in distilled water after a brown color appeared. The tissue sections were counterstained with hematoxylin and dehydrated in a graded ethanol series. Immunohistochemical staining was scored according to the product of the scores for the staining intensity and percentage of positively stained tumor cells. The staining intensity was scored as follows: 0 points (negative); 1 point (light brown); 2 points (brown); and 3 points (dark brown). The percentage of positively stained cells was scored as follows: 0 points (positive staining in less than 10% of cells), 1 point (10-25%), 2 points (26-50%), 3 points (51-75%) and 4 points (more than 75%). Total scores of <6 and ≥6 was defined as negative and positive, respectively.

**Immunofluorescence**

WT HLF and MHCC97H cells were fixed with 4% paraformaldehyde for 15 min prior to permeabilization with 0.5% Triton X-100 for 20 min at room temperature. Primary antibodies (0.2 µg/ml) were added for 2 hours at room temperature after blocking with 5% bovine serum albumin for 1 hr. DyLight549-conjugated goat anti-mouse IgG and DyLight649-conjugated goat anti-rabbit IgG were used as secondary antibodies. Nuclei were counterstained with DAPI. Images were acquired by confocal laser scanning microscopy on a Nikon Digital ECLIPSE C1 system (Nikon Corporation). For tissue immunofluorescence, we used the method of Tyramide signal amplification (TSA) to detect PD-1 and CD8 in mouse tissues. The preliminary sample processing steps are the same as IHC, and the subsequent steps are as follows: after incubating the anti-CD8 antibody (Servicebio, GB15068) at 4°C overnight, incubate with HRP-conjugated anti-rabbit secondary antibody at room temperature for 30 minutes, then incubate with IF488-Tyramide (Servicebio, G1231) at room temperature for 15 minutes, followed by antigen retrieval and other IHC steps, then incubate with anti-PD-1 antibody (CST, 84651), and after incubating with HRP-conjugated anti-rabbit secondary antibody the next day, use IF555-Tyramide (Servicebio, G1233), and finally stain the nuclei with DAPI.

**RNA-seq**

One group containing MHCC97H-shCon, MHCC97H-shSMAD3, and MHCC97H-shMETTL3 cells and another group containing HLF-Vec and HLF-ITIH1 cells were lysed with TRIzol reagent (Sigma). Then, RNA extraction, library preparation, transcriptome sequencing and data analysis were carried out by Novogene Company (Beijing, China).

**Ribonucleoprotein complex immunoprecipitation (RNP IP) assay**

Briefly, total cell lysates were immunoprecipitated with Dynabeads (Thermo Fisher) coated with protein A/protein G and precoupled to YTHDF2 (#71283S from CST) at 4 °C overnight. The pellets were washed three times with a buffer containing 50 mM Tris-HCl (pH 8.0), 150 mM NaCl, 0.5% Triton X-100, and 1× cOmplete (Roche). Total RNA was isolated from the immunocomplexes using QIAzol Lysis Reagent, reverse transcribed, and amplified by qPCR as described above.

**MS2-Flag RNA immunoprecipitation**

The pcDNA3.1+ plasmid was subjected to homologous ligation with the 6×MS2 sequence to construct the pcDNA3.1-6×MS2 plasmid. mRNA sequences containing the 5’ UTR (untranslated region) and 3’ UTR of ITIH1-wt and ITIH1-mut5 were inserted into the EcoRI and XbaI restriction sites in pcDNA3.1-6×MS2. Then, 10 μg of the pMS2-FLAG was cotransfected with the pcDNA-6×M2-ITIH1-wt or pcDNA-6×M2-ITIH1-mut5 plasmid into the corresponding HEK293 cells, and the cells were harvested 48 h later. Subsequently, the cell lysis buffer was precleared with 50 μl of protein G-agarose (Santa Cruz, American) at 4 °C for 2 h. The supernatant was then incubated with anti-Flag antibodies at 4 °C overnight with gentle shaking. This was followed by the addition of 50 μl of protein A/G-agarose for another 2 h. Finally, the beads were washed and resuspended in 50 μl of 2× SDS–PAGE loading buffer and boiled for 10 min before Western blot analysis.

**Luciferase reporter assay**

Luciferase activity was detected using a Dual-Luciferase Reporter Assay System (Promega, Madison, WI, USA) according to the manufacturer’s instructions. The relative luciferase activity was determined with a GloMax 20/20 Luminometer (Promega). Firefly luciferase activity was normalized to Renilla luciferase activity. The psiCHECK-2 plasmid contains two separate luciferase genes—firefly luciferase (as the reporter gene) and Renilla luciferase (as the reference gene). The gene of interest in this study, the whole CDS region with its 3' UTR of ITIH1, has been cloned into the 3' UTR of the firefly luciferase gene. If the m6A methylation enzymes interacts with the target sequence, it will lead to a decrease in firefly luciferase activity, which can be measured through a decrease in luminescence.

**m6A mRNA immunoprecipitation**

m6A mRNA immunoprecipitation was performed by first isolating poly(A)+ RNA from MHCC97H-shMETTL3 and MHCC97H-oeMETTL3 cells. Protein G Dynabeads (Thermo Fisher Scientific, Baltics UAB) were washed three times in 1 mL of IPP buffer (10 mM Tris-HCl (pH 7.4), 150 mM NaCl, and 0.1% NP-40). A total of 25 μl of beads was required for each IP reaction. An anti-N6-methyladenosine human monoclonal antibody (EMD Millipore, Temecula, CA, MABE1006) was added to the beads (5 μg/IP reaction) and the mixture was diluted in 1 mL of IPP buffer. The bead mixture was incubated overnight at 4 °C with rotation. The beads were washed five times with IPP buffer, and 100 ng of poly(A)+ RNA was added to the beads along with 1 mM DTT and RNase OUT. The mixture was diluted to 500 μl with IPP buffer. The bead mixture was incubated at 4 °C for 4 h with rotation. The beads were washed twice in IPP buffer, placed into a fresh tube, and washed more than three times in IPP buffer. m6A RNA was eluted from the beads by two rounds of incubation with 125 μl of 2.5 mg/mL N6-methyladenosine-5’-monophosphate sodium salt (CHEM-IMPEX INT’L INC., Wood Dale, IL) with rotation. TRIzol-LS was added to the supernatant, and RNA isolation was performed according to the manufacturer’s protocol. The final RNA sample was diluted in 10 μl of water.

**Flow cytometry**

The liver tumors of mice were isolated and then cut into small pieces, suspended in 1640 medium containing DNase I (Biosharp, BS137) and Collagenase IV (Biosharp, BS165), and further dissociated with gentleMACS Dissociator. The resulting suspension was filtered through a 70μm cell strainer and dissociation was terminated using FACS buffer (PBS containing 2% FBS). Red blood cells were removed by red blood cell lysis buffer, and the single-cell suspension was subjected to density gradient centrifugation at 400g for 30 minutes in 37% and 70% Percoll (Cytiva), with the leukocytes collected from the middle layer. The cells were then divided into two parts, with one part used for the detection of lymphocytes and myeloid cells (GR-1, NK1.1, CD19, LY-6G, CD11c, CD206, CD45, CD3, CD11b, LY-6C, F4/80, CD86), and the other part incubated in a 37°C culture incubator for 4 hours under cocktail (Leuko Act Cktl With GolgiPlug, BD, 550583) stimulation, followed by fixation and permeabilization (Transcription Factor Buffer Set, BD, 562574) for T cell detection (CD8a, PD-1, IFNγ, CD62L, CD69, CD45, CD3, CD44, CD4). The sources of the antibodies used for flow cytometry are as follows: GR-1, BD, 553126; NK1.1, BD, 553165; CD19, BD, 562291; LY-6G, BD, 560602; CD11c, BD, 558079; CD206, BD, 565250; CD45, BD, 557659; CD3, BD, 562600; CD11b, BD, 562950; LY-6C, BD, 563011; F4/80, BD, 743282; CD86, BD, 740877; CD8a, BD, 553030; PD-1, BD, 551892; IFNγ, BD, 560660; CD62L, BD, 560516; CD69, BD, 560689; CD44, BD, 563114; CD4, BD, 563151.

**Cell line and cultures**

The human embryonic kidney cell line HEK293, human normal liver cell line HL7702, human hepatoma cell line HepG2, and HCC cell lines Hep3B, ALEX and HLF were purchased from the China Center for Type Culture Collection (CCTCC, Wuhan, China). The HCC cell lines MHCC-MHCC97H and HCC-LM3 were obtained from the Liver Cancer Institute, Zhongshan Hospital, Fudan University, Shanghai, China. All cell lines were cultured in Dulbecco’s modified Eagle’s medium (DMEM) supplemented with 10% fetal bovine serum (Gibco, Grand Island, NY, USA) and maintained at 37 °C in a 5% CO2 incubator. Lentivirus-producing cells, such as Hep3B-shITIH1, HLF-oeITIH1, and MHCC97H-oeITIH1 cells, were grown in the same medium supplemented with 2 μg/ml puromycin and penicillin (100 U/ml)-streptomycin (100 μg/ml).

**Microcomputed tomography (micro-CT)**

We used CT scanning to evaluate the effectiveness of drug therapies for HCC. At the end of the treatment phase, mice were subjected to abdominal scanning on a MICRO-CT SKYSCAN 1276 (Bruker, Germany) at the Institute of Hydrobiology of the Chinese Academy of Sciences. Each mouse was anesthetized with 2% isoflurane followed by 200 μl of contrast material (Iopromide, Ultravist 370, Bayer Vital, Germany) via the tail vein injection before scanning and was quickly placed in the scanning bed for scanning and image reconstruction.

**FRAP**

MHCC-97H cells transfected with EGFP-METTL3 by using Lipofecamine 3000 (L3000001, Invitrogen) according to the manufacture’s instructions. Post-transfection, fluorescence recovery after photobleaching (FRAP) assays were conducted on an Olympus FV3000 Confocal Laser Scanning Microscope. 24 hours after transfection, METTL3 condensates were subjected to photobleaching using a 40% intensity 488nm laser. The recovery of fluorescence was monitored over a defined period. The mean fluorescence intensity was subsequently quantified using ImageJ software.

**Fusion and fission**

MHCC-97H cells transfected with EGFP-METTL3 using the Lipofectamine 3000 reagent (Catalog No. L3000001, Invitrogen), following the manufacturer's protocol. After 24 h, cells were imaged on Olympus FV3000 Confocal Laser Scanning Microscope.

**Supplementary** **Figures**

**
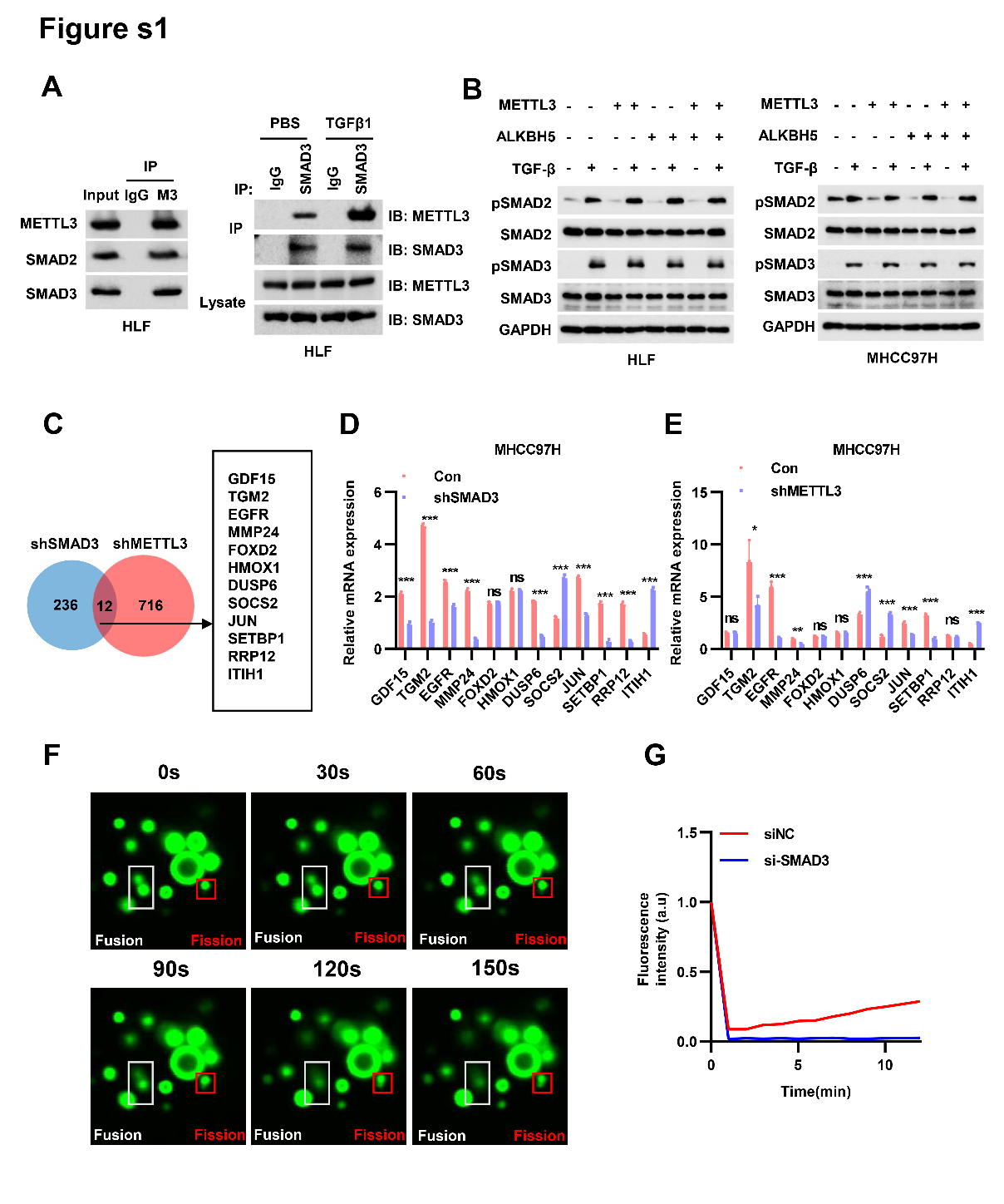
**

**Figure S1. The correlation between METTL3, SMAD3, and ITIH1. (A)** The results of METTL3 and SMAD2/3 co-IP and the results of SMAD3 and METTL3 endogenous co-IP with or without TGF-β stimulation for 12h in HLF cells**. (B)** The levels of activated and total SMAD2/SMAD3 were determined through WB in HLF cells and MHCC97H cells transfected with METTL3 and ALKBH5 and then stimulated with TGF-β for 12 h. **(C)** The intersection of two RNA-seq datasets, MHCC97H-Con vs. MHCC97H-shSMAD3 and MHCC97H-Con vs. MHCC97H-shMETTL3. **(D)** The relative mRNA expression levels of the overlapping genes identified in MHCC97H-Con and MHCC97H-shSMAD3 cells. **(E)** The relative mRNA expression levels of the overlapping genes identified in MHCC97H-Con and MHCC97H-shMETTL3 cells. **(F)** GFP-METTL3 puncta undergo fusion and fission, images are shown at indicated timepoints. **(G)** Quantification of fluorescence intensity recovery of GFP-METTL3 in the photobleaching.


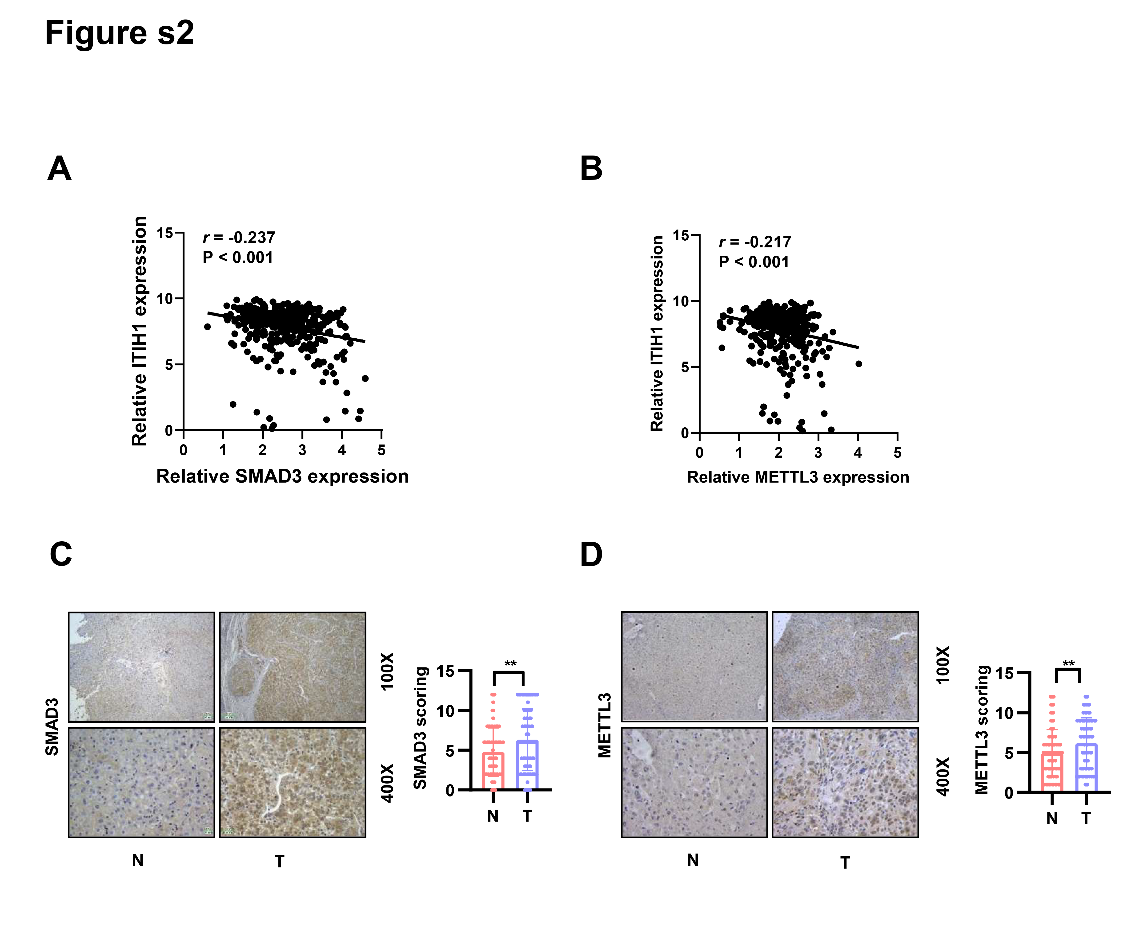


**Figure S2. The correlation between METTL3, SMAD3, and ITIH1. (A-B)** The correlations between ITIH1 and METTL3 expression and ITIH1 and SMAD3 expression as predicted by GEPIA analysis. **(C-D)** Representative results of IHC staining for SMAD3/METTL3 in paraffin-embedded HCC samples from Tongji Hospital. The statistical results of their expression in nontumor tissues (N) and tumor tissues (T) are presented in the right panel.


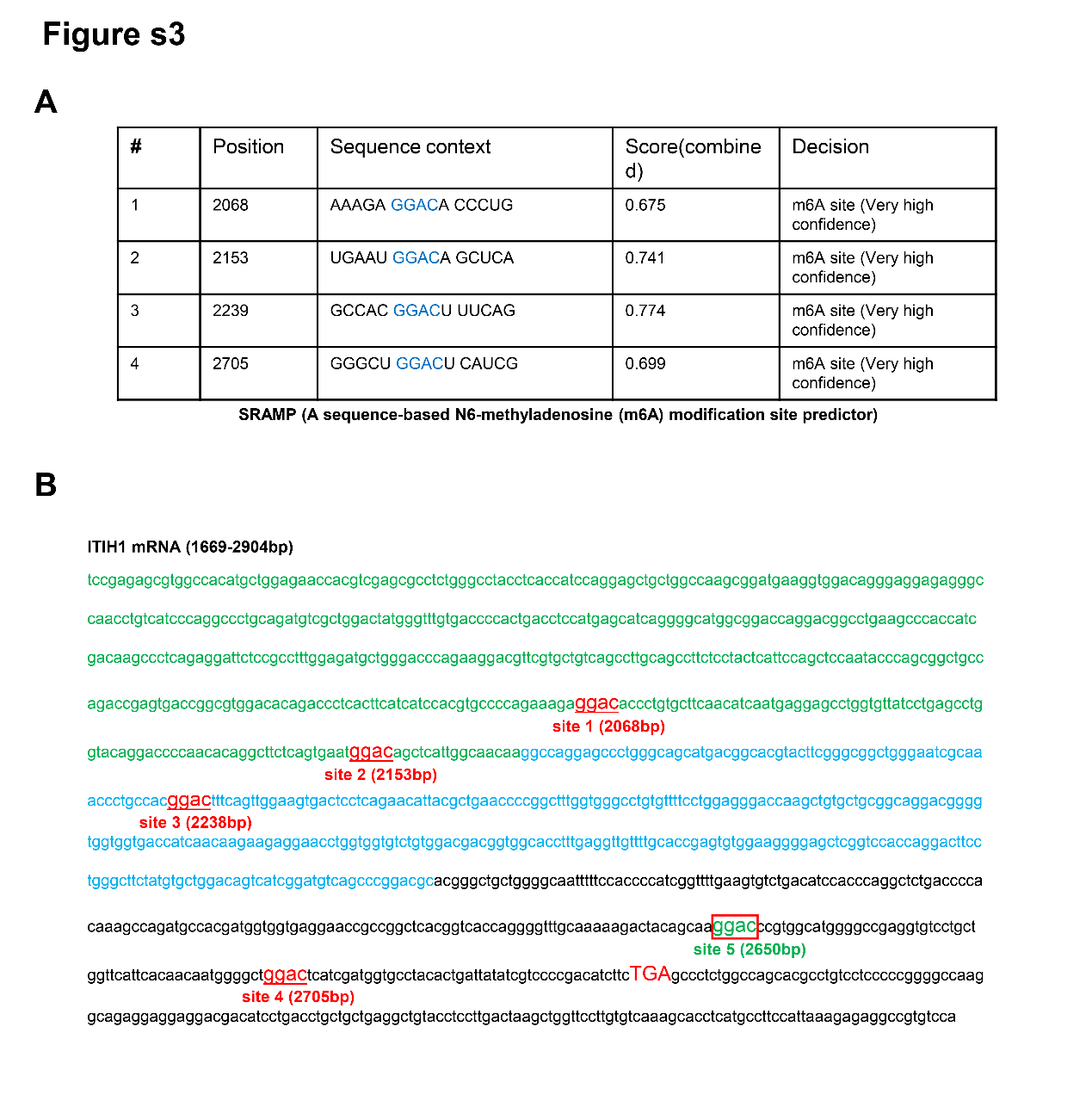


**Figure S3. The predicted m6A sites in ITIH1. (A)** The m6A sites in ITIH1 mRNA predicted by SRAMP. **(B)** The possible m6A sites in ITIH1 mRNA are indicated.


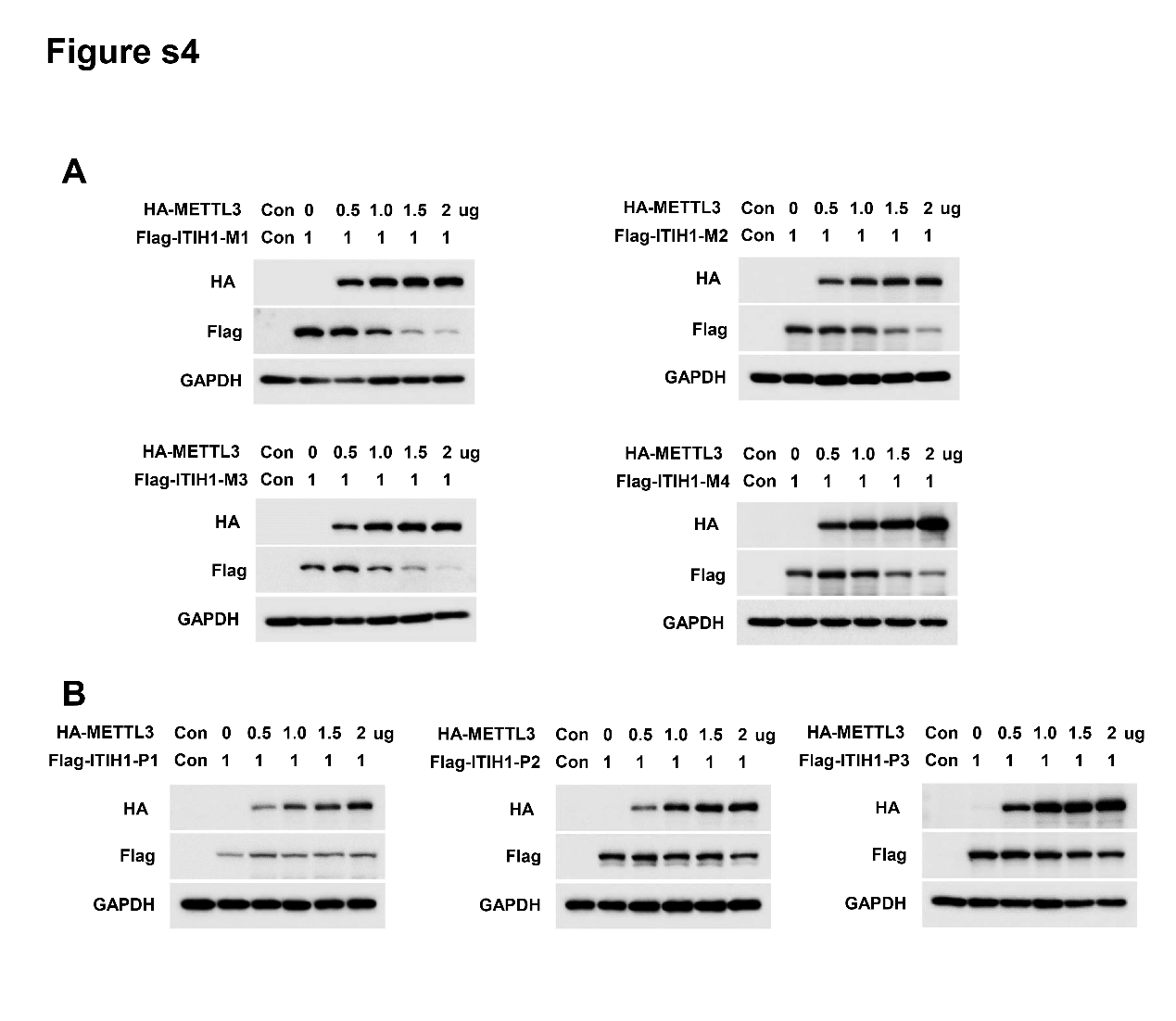


**Figure S4. WB verification of the m6A sites in ITIH1. (A)** The protein levels in cells cotransfected with HA-METTL3 and Flag-ITIH1-M1/M2/M3/M4 in a concentration-dependent manner. **(B)** The protein levels in cells cotransfected with the HA-METTL3 and ITIH1 truncation mutant plasmids in a concentration-dependent manner.


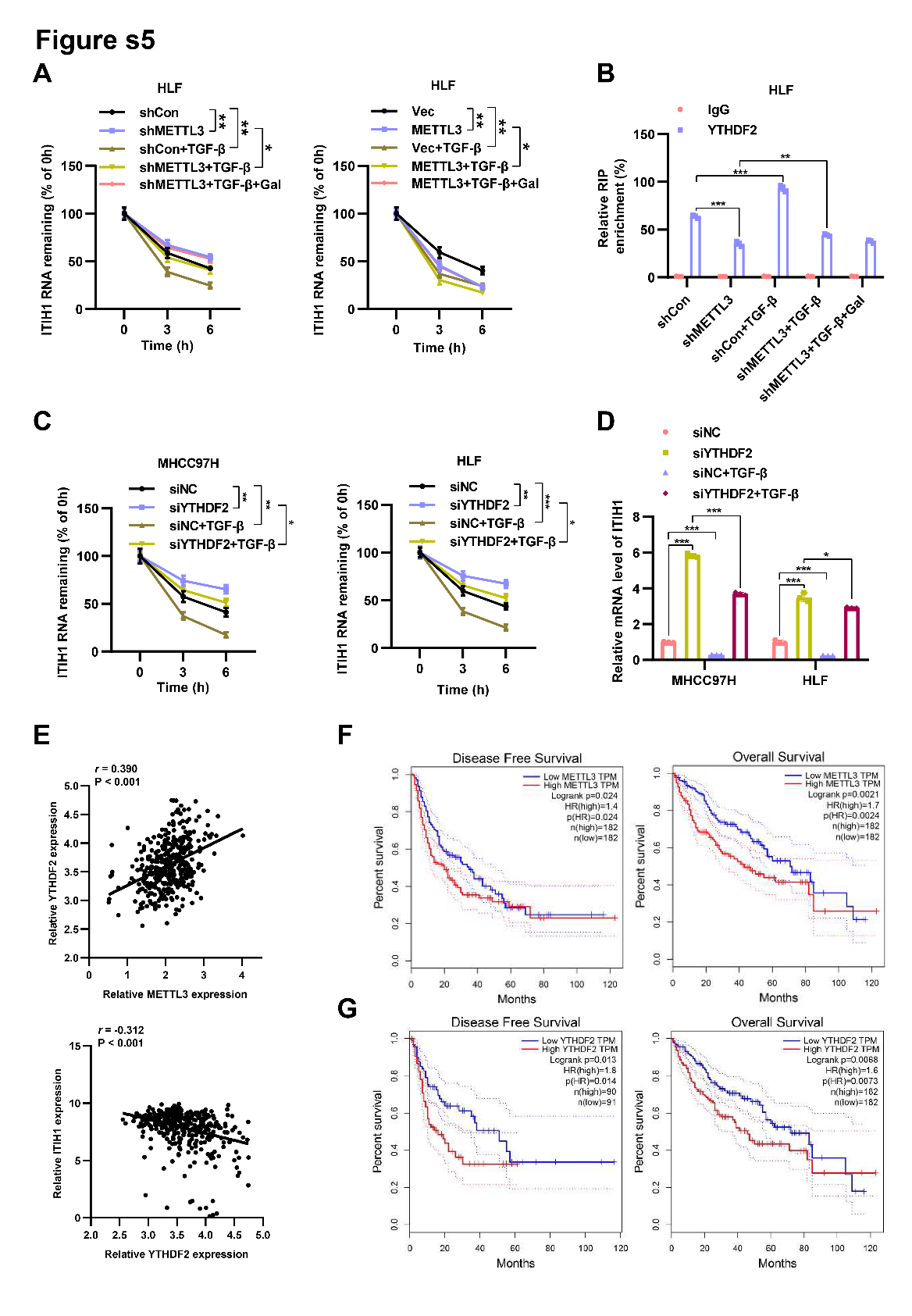


**Figure S5. The correlations between YTHDF2/METTL3 expression and YTHDF2/ITIH1 expression. (A)** Levels of remaining ITIH1 mRNA in HLF-shMETTL3 and HLF-METTL3 cells treated with TGF-β, galunisertib, and actinomycin D for the indicated times. **(B)** Relative ITIH1 levels in HLF-shMETTL3 cells treated with TGF-β or galunisertib as determined by RIP with an anti-YTHDF2 antibody. **(C)** Levels of remaining ITIH1 mRNA in HLF-siYTHDF2 and MHCC97H-siYTHDF2 cells treated with TGF-β, galunisertib, and actinomycin D for the indicated times. **(D)** The relative ITIH1 mRNA expression in HLF-siYTHDF2 and MHCC97H-siYTHDF2 cells treated with TGF-β. **(E)** The correlation between METTL3, ITIH1 and YTHDF2 expression from GEPIA. **(F)** The DFS and OS of patients with low or high METTL3 expression from GEPIA. **(G)** The DFS and OS of patients with low or high YTHDF2 expression from GEPIA.


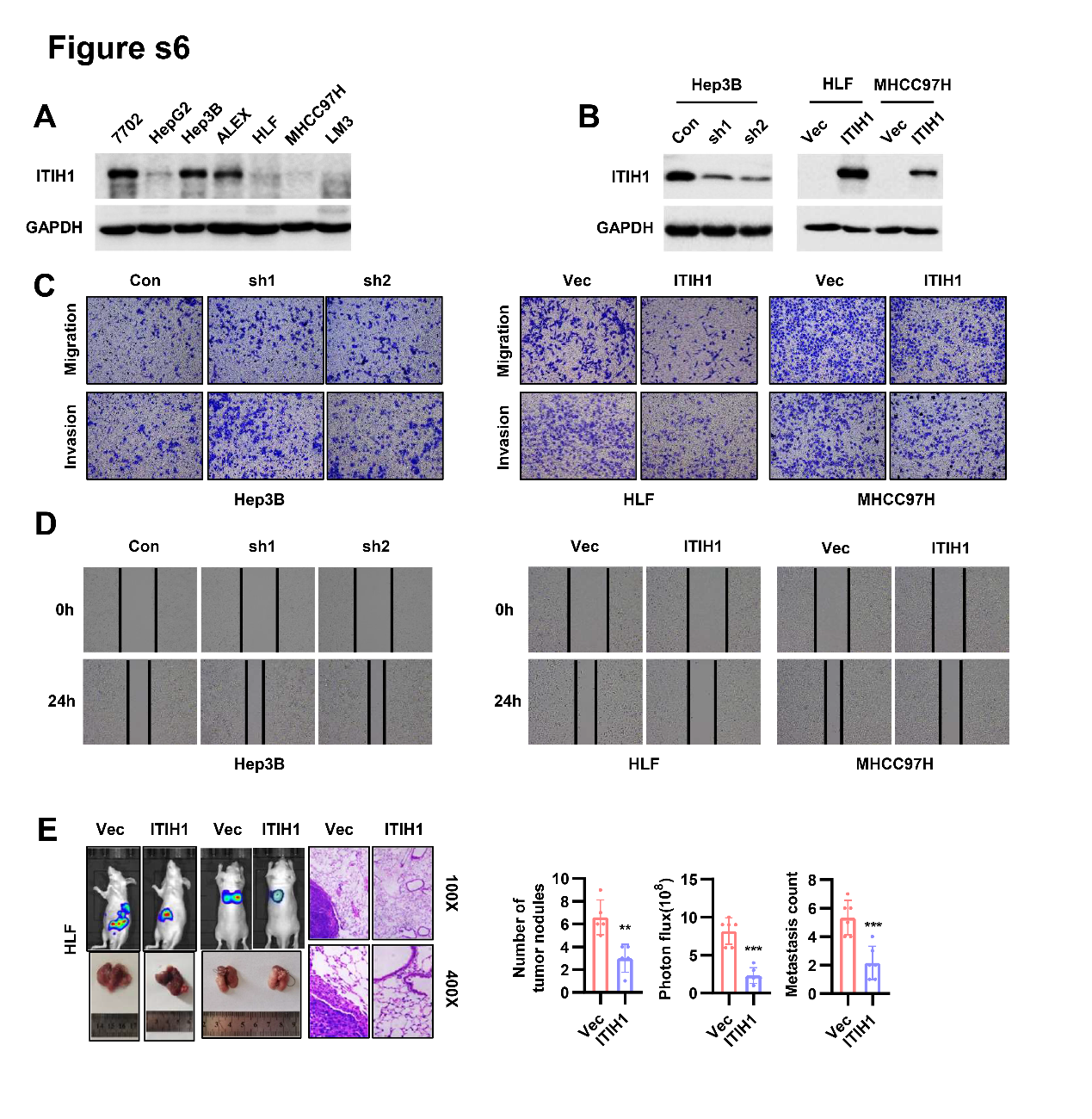


**Figure S6. The effects of ITIH1 *in vitro and in vivo*. (A)** The ITIH1 protein levels in HCC cell lines were examined through WB. **(B)** Verification of ITIH1 levels in Hep3B-shITIH1, HLF-oeITIH1, and MHCC97H-oeITIH1 cells. **(C-D)** The wound healing assay results and Transwell assay results in the indicated cell lines. **(E)** The results of the orthotopic transplantation model and tail vein injection model in nude mice established using HLF-luc-ITIH1 or vector cells. The statistical results are presented in the right graphs. Representative image of H&E staining of lung metastases.


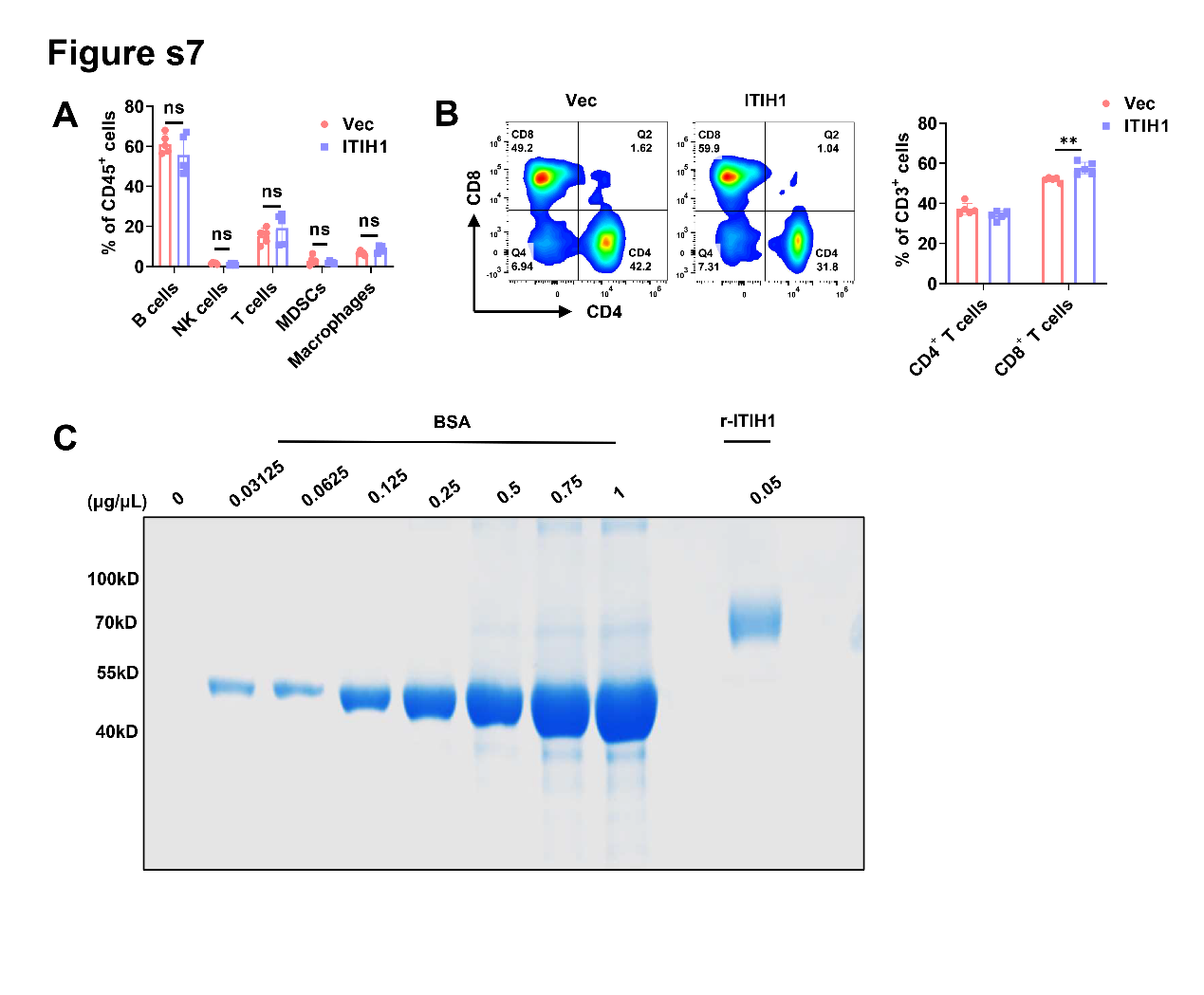


**Figure S7. Effect of ITIH1 on immune microenvironment of HCC and purification of ITIH1 recombinant protein. (A)** The proportion of various immune cell subsets in tumors in control and overexpressed ITIH1 groups was measured by flow cytometry. **(B)** Flow cytometry was used to detect the proportion of CD8 T^+^ cells in the tumors of control and overexpressed ITIH1 groups. **(C)** The Coomassie brilliant blue staining of r-ITIH1.


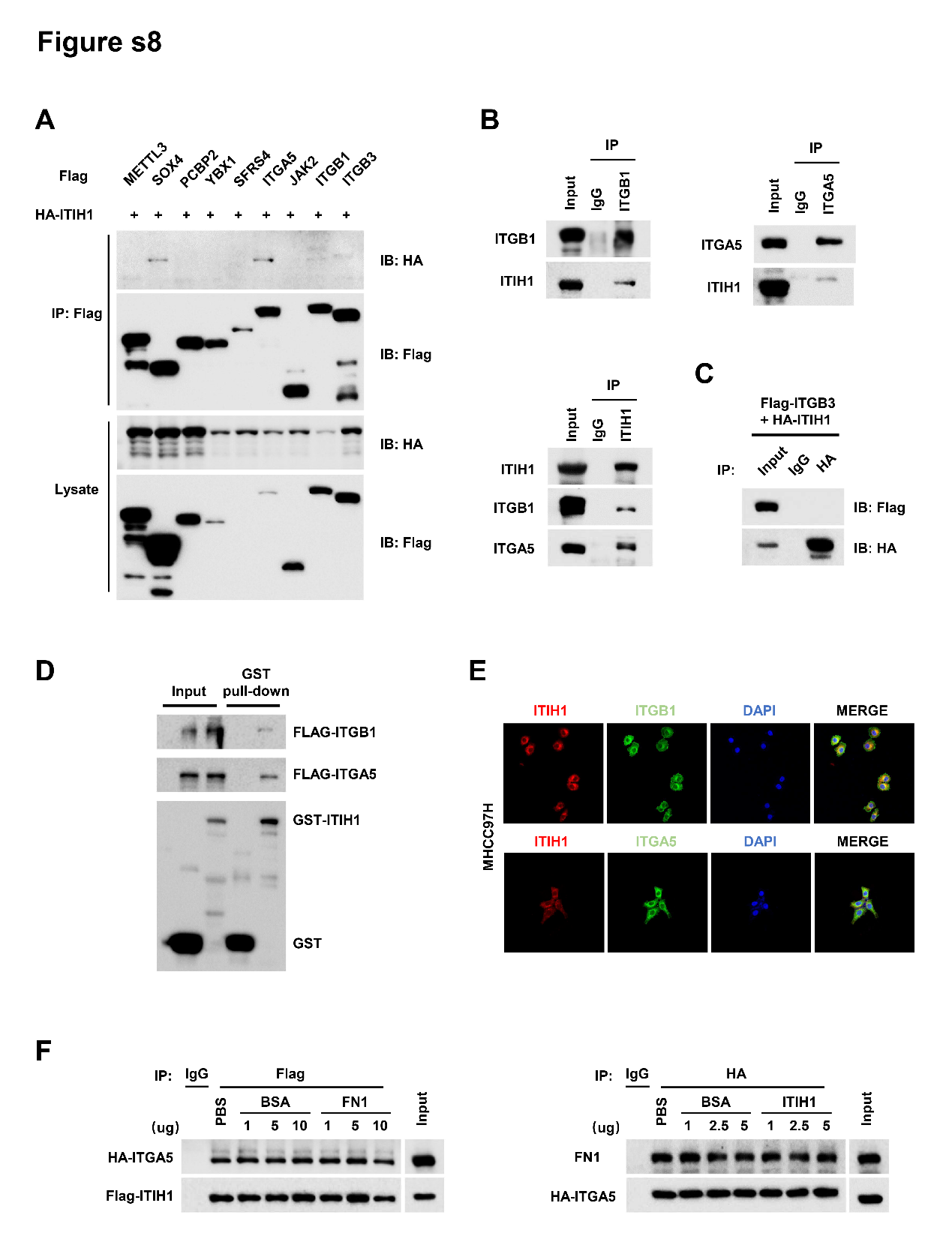


**Figure S8. Verification of the interactions between ITIH1 and integrin family members. (A)** The results of IP with HA-ITIH1 and other indicated proteins in 293T cells. **(B)** The results of IP with ITIH1, ITGB1, and ITGA5 in HLF cells. **(C)** The results of IP with ITIH1 and ITGB3 in 293T cells. **(D)** The results of pull-down with GST-ITIH1 and other FLAG labelled proteins. **(E)** Confocal images of IF staining for ITIH1+ITGB1 and ITIH1+ITGA5 in MHCC97H cells. **(F)** The results of IP with ITIH1+ITGA5 and ITGA5+FN1 when FN1 or r-ITIH1 was added in a concentration gradient.


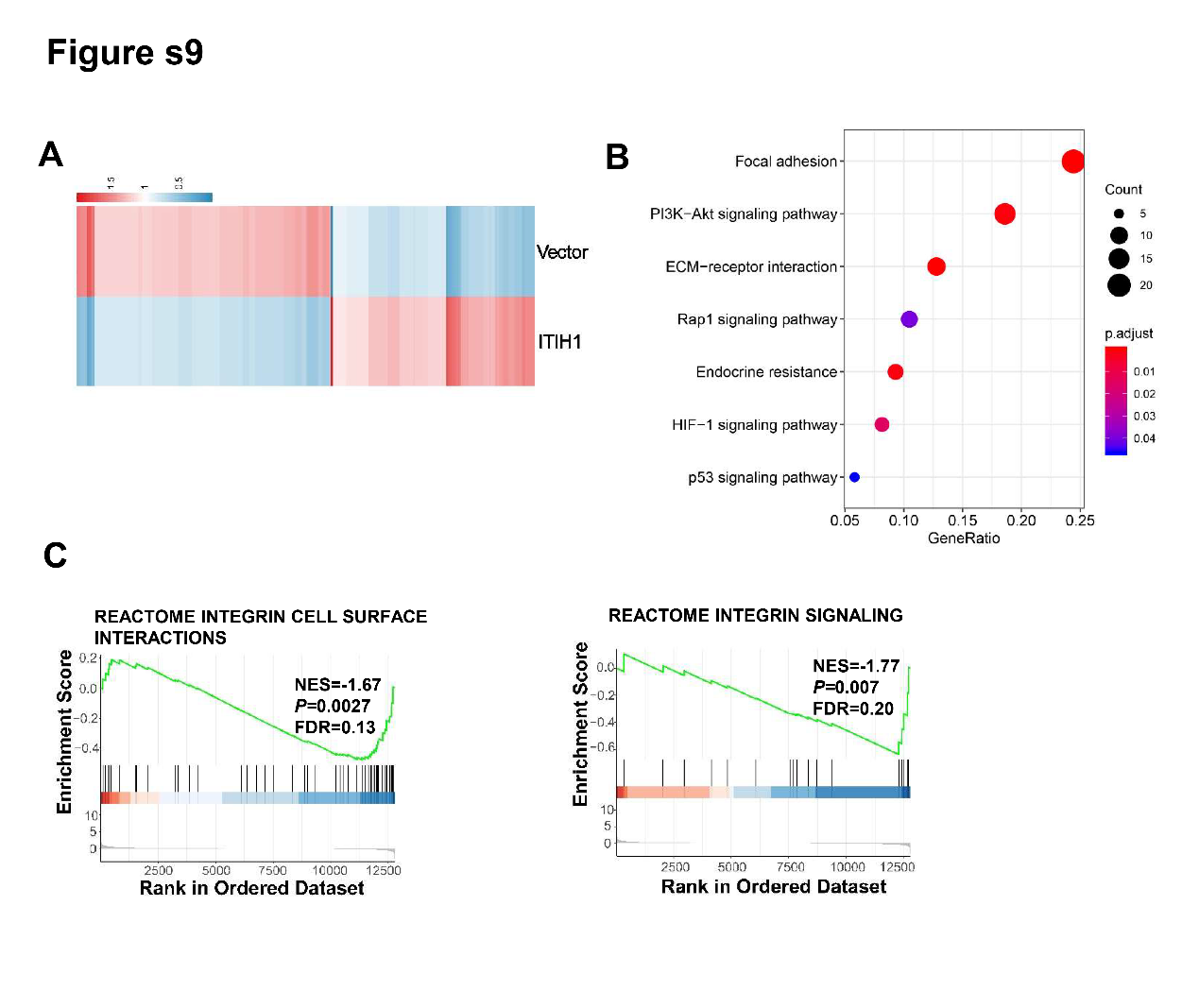


**Figure S9. Bioinformatics analysis of cells with ITIH1 overexpression. (A)** Heatmap representation of RNA-seq data showing the expression of all differentially expressed genes in ITIH1-overexpressing and control- HLF cells. **(B)** KEGG enrichment analysis of all downregulated genes in the ITIH1-overexpressing group. **(C)** Gene set enrichment analysis showed that integrin signaling pathway-related genes in the Reactome database were significantly enriched in control HLF cells compared with HLF cells with ITIH1 overexpression.


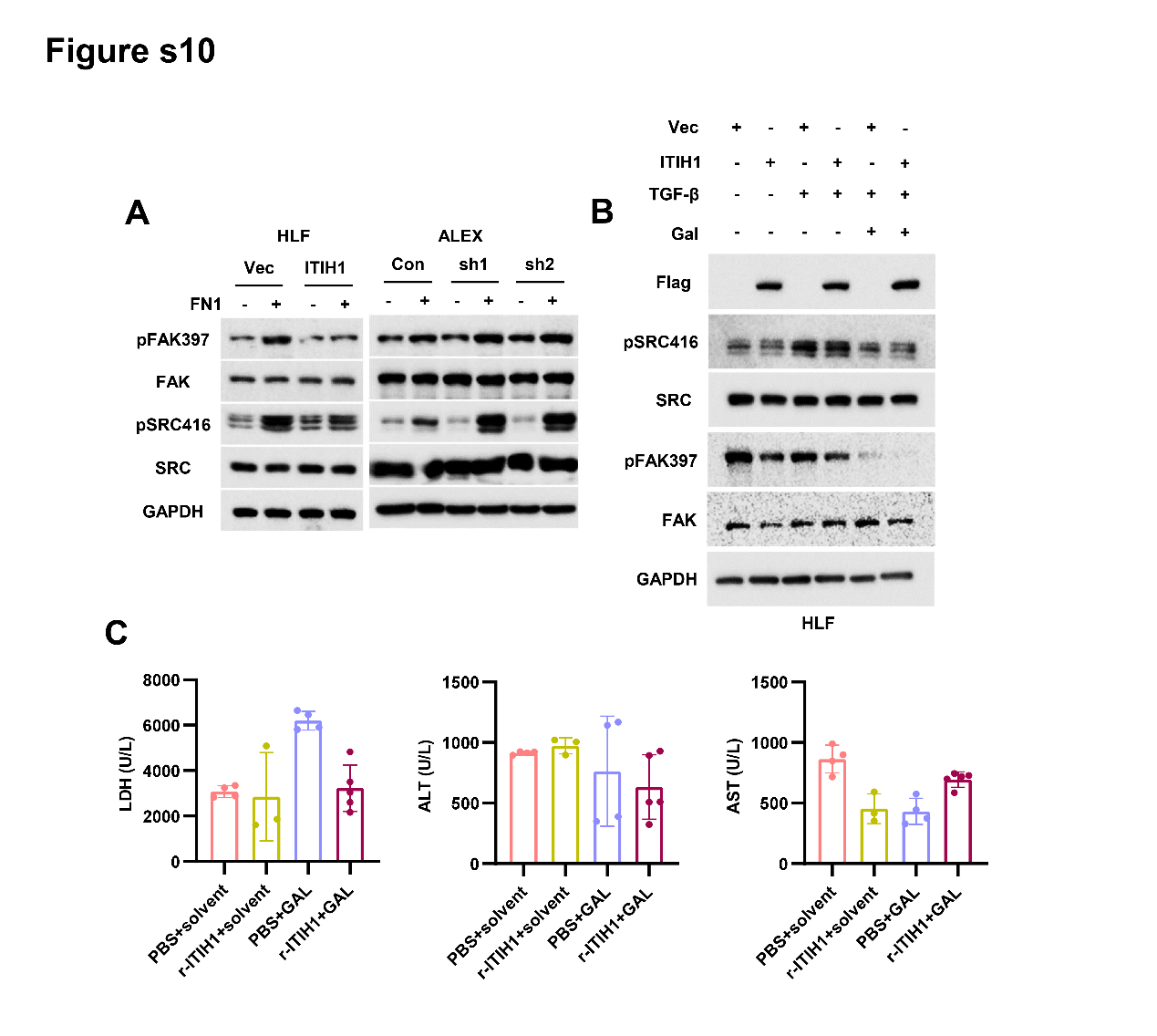


**Figure S10. Changes in signaling pathways caused by ITIH1 and the in vivo effects of ITIH1. (A)** HLF-oeITIH1 and ALEX-shITIH1 cells were treated with FN1 stimulation for 2 hr. Then, the cells were collected for WB analysis. **(B)** HLFs were transfected with ITIH1 and then treated with TGF-β and Gal alone or in combination. Then, the cells were analyzed by Western blotting. **(C)** Before sacrifice, the blood of C57 mice described in Fig. 6F was collected and subjected to each analysis.


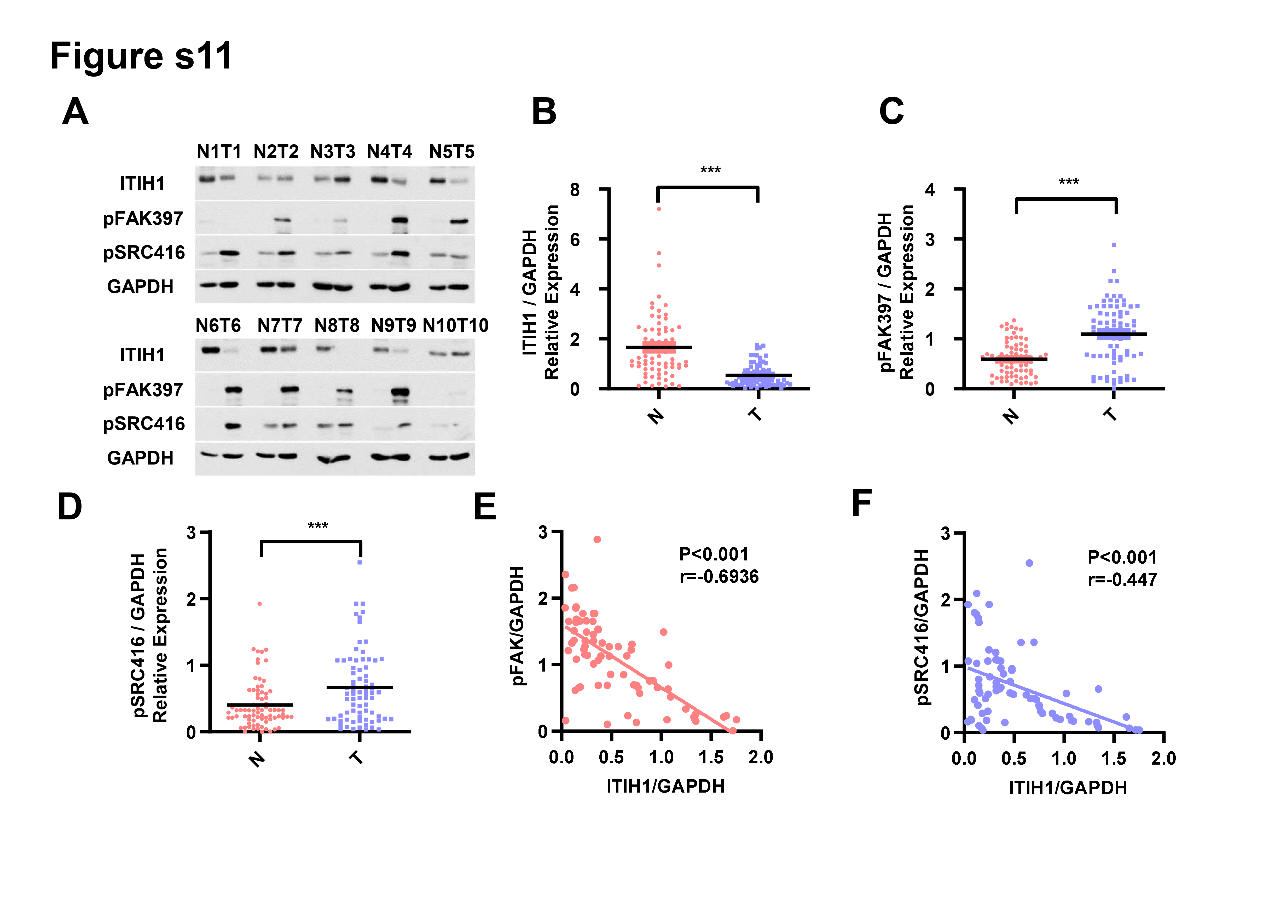


**Figure S11. The correlations between the protein levels of ITIH1 and pFAK/pSRC. (A)** WB analysis of HCC patients from Tongji Hospital. We analyzed the relative expression of ITIH1. **(B-D)** We analyzed the relative protein levels of ITIH1, pFAK, and pSRC in nontumor tissues and tumor tissues. **(E-F)** The statistical correlations between the levels of pFAK/ITIH1 and pSRC/ITIH1.
